# Using net-zero carbon debt to track climate overshoot responsibility

**DOI:** 10.1073/pnas.2409316122

**Published:** 2025-03-24

**Authors:** Setu Pelz, Gaurav Ganti, Robin Lamboll, Luke Grant, Chris Smith, Shonali Pachauri, Joeri Rogelj, Keywan Riahi, Wim Thiery, Matthew J. Gidden

**Affiliations:** ^a^International Institute for Applied Systems Analysis, Energy Climate and Environment Program, Laxenburg A-2361, Austria; ^b^Potsdam Institute for Climate Impact Research, Climate Economics and Policy - MCC Berlin Research Department, Member of the Leibnitz Association, Potsdam D-14412, Germany; ^c^Integrative Research Institute on Transformations of Human-Environment Systems, Humboldt-Universität zu Berlin, Berlin D-10099, Germany; ^d^Geography Department, Humboldt-Universität zu Berlin, Berlin D-10099, Germany; ^e^Climate Analytics, Berlin D-10969, Germany; ^f^Centre for Environmental Policy, Grantham Institute for Climate Change and the Environment, Imperial College London, London SW72AZ, United Kingdom; ^g^Grantham Institute for Climate Change and the Environment, Imperial College London, London SW72AZ, United Kingdom; ^h^Department of Water and Climate, Vrije Universiteit Brussel, Brussels, Belgium

**Keywords:** overshoot, carbon debt, intergenerational equity, interregional equity, impacts

## Abstract

Current emissions trends will likely deplete a 1.5 °C consistent carbon budget around the year 2030, resulting in at least a temporary exceedance, or overshoot. To clarify responsibilities for this budget exceedance, we consider “net-zero carbon debt,” a forward-looking measure of the extent to which a party is expected to breach its “fair share” of the remaining budget by the time it achieves net-zero carbon emissions. We apply this measure to all vetted mitigation scenarios assessed in the Intergovernmental Panel on Climate Change’s Sixth Assessment Report and two scenarios that model current policies and pledges, using an illustrative equal per capita allocation of a remaining 1.5 °C carbon budget starting in 1990. The resulting regional carbon debt estimates inform i) the scale and pace of regional carbon drawdown obligations necessary to address budget exceedance and ii) relative regional responsibilities for increased lifetime exposure to extreme heatwaves across age cohorts due to budget exceedance. Our work strengthens intergenerational equity considerations within an international climate equity discourse and informs the implementation of effort-sharing mechanisms that persist beyond the exhaustion of a rapidly dwindling remaining carbon budget.

## Significance

Exceeding the Paris Agreement’s 1.5 °C limit raises urgent questions of how countries’ responsibilities for climate action change in this new context. We introduce “net-zero carbon debt” as a forward-looking measure of responsibility for surpassing the remaining carbon budget at net-zero carbon emissions, drawing a line at the warming threshold, and clarifying obligations to restore temperatures below it. By applying this to future scenarios, we show how regional debts may accrue and use these estimates to allocate responsibilities for the heightened extreme heatwaves and drawdown burdens younger generations will inherit. Our findings reveal how the lack of global cooperation prolongs climate harms. Moreover, they show how responsibilities persist beyond budget exhaustion, informing the need for for increased international support, ambitious mitigation targets, and reparative measures for those most affected.

The first Global Stocktake of progress under the Paris Agreement issued a clear warning: Near-term global greenhouse gas (GHG) emissions trends are not aligned with modeled pathways consistent with limiting warming to 1.5 °C (1). Continued failure to reduce emissions in line with global climate objectives will increase the frequency and magnitude of extreme climate events, alongside impacts already experienced by many communities around the world and threatening the achievement of sustainable development objectives in developing regions (2). The injustice inherent in these differentiated impacts and vulnerabilities is amplified by the uneven distribution of responsibilities for historical and future climate change (3–6).

Scientists and analysts combine normative assessments of mitigation efforts with physical science evidence to inform policy deliberations aimed at addressing these issues. A group of these assessments informs the distribution of efforts, or “fair shares,” to cut emissions based on a range of principled considerations, drawing on a rich body of literature spanning decades, discussed at length in the Intergovernmental Panel on Climate Change Working Group III assessment reports (e.g., refs. 7 and 8). Other equally important assessments include adaption efforts and loss and damage contributions, both informed by scientific and normative considerations (9).

Despite the guidance provided by assessments of fair shares, pledged emission reduction targets in Nationally Determined Contributions (NDCs) continue to fall short of appropriately considering the foundational principles of equity and common but differentiated responsibilities and respective capabilities enshrined in the United Nations Framework Agreement on Climate Change and the Paris Agreement (10, 11). This shortfall is compounded by the failure to translate (insufficient) targets to action, with recent research finding that current NDCs and Long-term Low Emissions Development Strategies are not only misaligned with climate objectives but also lack credible implementation evidence (12).

As a result of insufficient action to date, contemporary scientific and normative guidance aimed at informing climate deliberations must grapple with a dwindling 1.5 °C aligned remaining carbon budget (RCB), which recent estimates place at approximately 200 Gt CO_2_ for a 50% chance of staying below 1.5 °C from the year 2024 (13). Shrinking RCBs are a challenge to notions of fair shares which typically rely on a “remaining” quantity to distribute. Under the 1.5 °C limit, this quantity is expected to expire within the next 10 y even under the most ambitious mitigation pathways. This stark physical reality leaves us at a crossroads: either relax the temperature limit to create a larger budget for distribution or evolve our approach to fair shares to reconcile with a depleted budget. We contend that relaxing the temperature limit is inherently inequitable, disproportionately harming vulnerable populations and increasing the likelihood of irreversible climate impacts (14). Instead, we advocate for the evolution of fair share approaches to account for and minimize budget exceedance, while equitably allocating responsibilities for temperature drawdown to below 1.5 °C as quickly as possible. In alignment with the literature to date, such approaches cannot reset the slate at budget depletion but must preserve responsibilities for previous contributions to climate change.

Recent work has begun to explore the science and policy dimensions of temporary budget exceedance, or overshoot, and what fair efforts mean in this case to keep limiting warming to 1.5 °C within reach. This work spans research on the estimation of excessive national emissions relative to fair shares (15, 16), fair allocations of national carbon dioxide removal (CDR) responsibilities (17, 18), the risk of high overshoot of the 1.5 °C limit in the absence of international cooperation (19) and the inter- and intragenerational inequities in the effects of higher global average temperatures under “peak-and-decline” budget overshoot pathways (20, 21). A strand of this literature specifically warns of the political, legal, and technological challenges associated with speculative late-century CDR to compensate for inadequate historical and near-term mitigation effort (22–25). Forward-looking measures of fair shares that can reconcile budget overshoot must address both intergenerational and international equity concerns this literature raises.

**Fig. 1.**
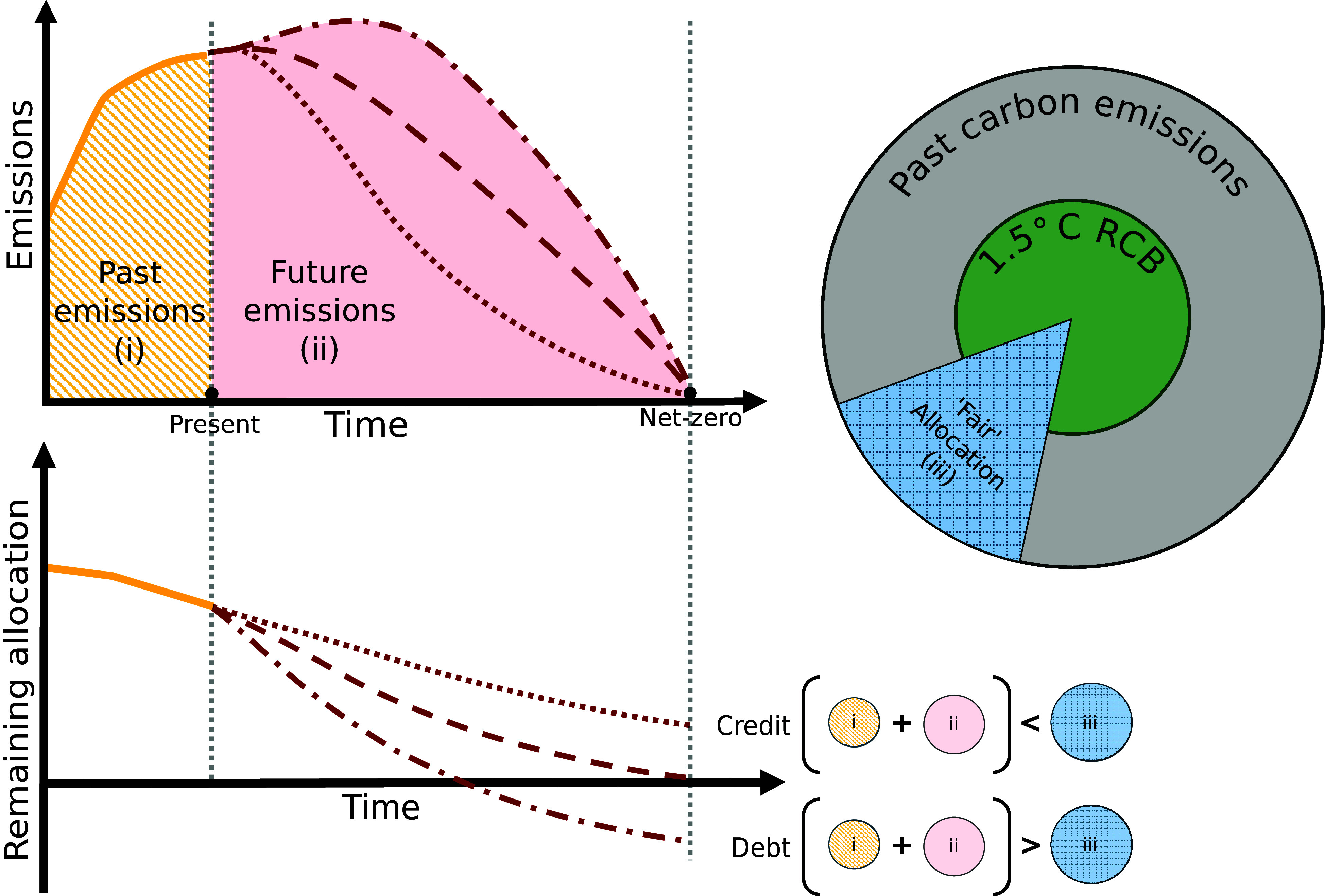
The persistent accrual of net-zero carbon debt. Regional net-zero carbon debt is quantified by subtracting (i) past and (ii) future cumulative carbon emissions from (iii) a fair allocation of the total carbon budget comprising both a RCB and past global carbon emissions as desired.

In this study, we consider “net-zero carbon debt,” a measure quantifying expected contribution to climate overshoot. We define this as an extension of historical carbon debt, proposed by Matthews and Gignac (15, 16), using modeled future emissions trajectories. The net-zero carbon debt measure considers three factors: i) past carbon emissions, ii) future carbon emissions, and iii) “fair” allocations of a RCB (Fig. 1). These factors quantify the carbon debt (or credit) accumulated by a party over a given period, where this debt peaks in the year the party achieves net-zero carbon emissions, which we term the net-zero carbon debt. We illustrate this measure by considering cumulative carbon emission from the fossil-fuel and industry sector (CO_2_-FFI) and, an equal cumulative per capita allocation of a 1.5 °C RCB (50% likelihood) from the year 1990. This allocation approach distributes the total carbon budget from the year 1990 equally to the global population over the years 1990-2050. Methodological and normative decisions taken for this illustrative assessment are discussed at length in our *Methods* section and in *SI Appendix*, alongside examples of other possible allocation approaches. While in this work, we quantify debt accrual at the regional level due to the available spatial resolution of global full-century emissions projections, the measure can be applied at any resolution with assumptions for the rest of the world.

In the remainder of this article, we present two applications of the net-zero carbon debt measure. First, drawing on the IPCC WGIII AR6 scenarios database, we calculate how regional net-zero carbon debts and corresponding carbon drawdown obligations vary under different regional net-zero timings. Second, we introduce two scenarios that represent current policies and pledges and compare the resulting regional carbon drawdown obligations with regional increases in age-cohort-specific lifetime extreme heatwave exposure. Together, these analyses underscore that equity considerations persist after the 1.5 °C RCB is exhausted, revealing stark interregional and intergenerational disparities in the responsibilities, impacts, and burden of net-zero carbon debt.

## Results

### Regional Carbon Debt Accrual in the IPCC’s WGIII AR6 Scenarios Database.

**Fig. 2.**
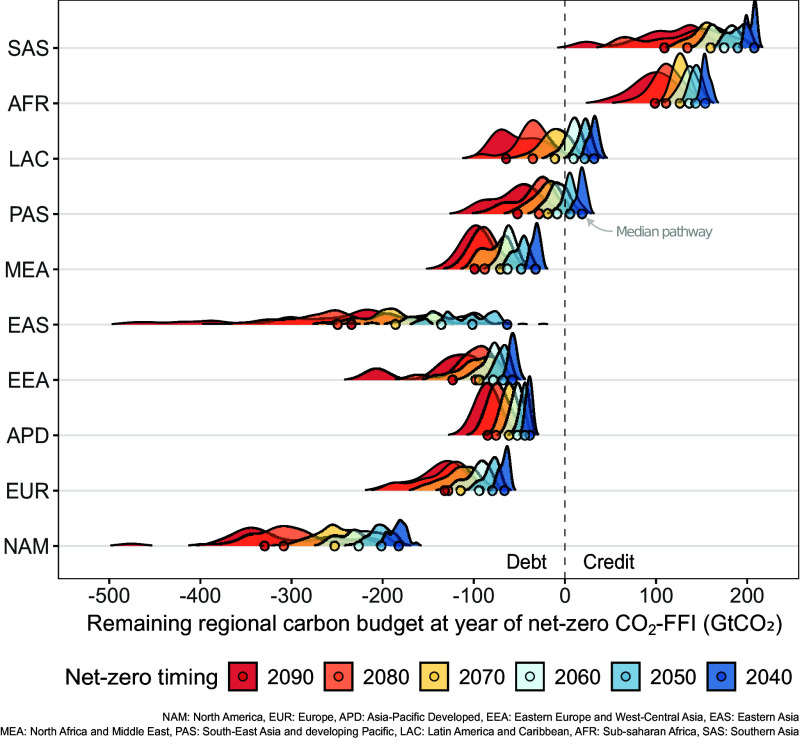
Assessing carbon debts accrued under varying regional net-zero CO_2_-FFI timings. Density plots describe scenario distributions of regional net-zero carbon debt accrual grouped by regional net-zero CO_2_-FFI timings in the AR6 scenarios database, subset to reflect a maximum 2090 regional net-zero CO_2_-FFI timing group. The circular shapes indicate the median net-zero carbon debt within each timing group.

We begin with the IPCC AR6 WGIII scenarios database (26, 27), focusing on scenarios where all regions achieve net-zero CO_2_-FFI by the end of the century. Using the net-zero carbon debt measure, we quantify the distribution of carbon debt accrual across scenarios and group the results by 10-y intervals of each region’s decadal net-zero timing (Fig. 2). The spread in our results illustrates the relationships between regional carbon debt accrual, past emissions, and future net-zero timings. We find three broad groups^*^ across the assessed scenarios: regions that consistently accrue net-zero carbon debt (i.e., are always responsible for overshoot regardless of their net-zero timing), regions that may accrue debt with later net-zero CO_2_-FFI timing, and regions that do not accrue debt this century. Regions comprising the first group include North America (NAM), Western Europe (EUR), Asia-Pacific Developed (APD), Eastern-Europe and West-Central Asia (EEA), Eastern Asia (EAS), and the Middle East (MEA). Regions comprising the second group include the Developing Pacific (PAS) and Latin America and the Caribbean (LAC), which only accrue net-zero carbon debt for net-zero CO_2_-FFI targets from 2050 onward. Regions comprising the third group include Sub-Saharan Africa (AFR) and Southern Asia (SAS). While magnitudes vary, this grouping persists across all allocation approaches examined in this study (*Methods* and *SI Appendix*, section 4).

The persistent accrual of net-zero carbon debt shown here raises climate fairness issues on multiple fronts. Across regions, it implies that some may need to counterbalance the excess emissions of others to keep global climate objectives within reach. Within regions, it implies that the burden of drawing down accrued carbon debt may be placed primarily on younger generations. This is particularly acute in regions expected to accrue large net-zero carbon debts even under high-ambition scenarios. For instance, under median regional pathways achieving net-zero CO_2_-FFI by the year 2050, net-zero carbon debt in the NAM and EAS regions reaches −201.4 GtCO_2_ and −101.2 GtCO_2_, respectively. Decisions on timeframes for drawing down these substantial debts affect how and by whom this effort is undertaken and will likely require the consideration of both increased global mitigation efforts through international cooperation and investment in additional permanent carbon dioxide removal (*SI Appendix*, section 4). This raises important questions on the use of the net-zero carbon debt measure to define relative regional carbon drawdown obligations, as the cooling effect of a unit of net-negative emissions may not be the exact opposite of the warming effect of a unit of gross emissions (28–31). To address this, we conduct experiments using the simple climate model FaIR (32), finding evidence for an approximate 1:1 long-term temperature equivalence between gross carbon emissions prior to net-zero and permanent net-negative carbon sequestration thereafter in scenarios that limit warming to 1.5 °C with no or limited overshoot, with a 25% uncertainty in either direction (*SI Appendix*, section 7). Notwithstanding important Earth System uncertainties that warrant further attention, this work provides initial support for the quantification of net-zero carbon debts and their translation into implied carbon drawdown obligations. The results show how these measures could support the assessment of interregional and intergenerational equity considerations in scenarios of future mitigation effort, informing how, when, and by whom carbon drawdown burdens must be met to minimize overshoot magnitude and duration. To explore the implications of net-zero carbon debt accrual and carbon drawdown obligations in the context of current global climate action, we now examine two scenarios representing alternative futures: one in which global mitigation effort continues to follow current policies (CurPol) and one in which all targets and pledges are achieved (CurPledge).

### Overshoot Responsibilities and Implications under Current Policies and Pledges.

**Fig. 3.**
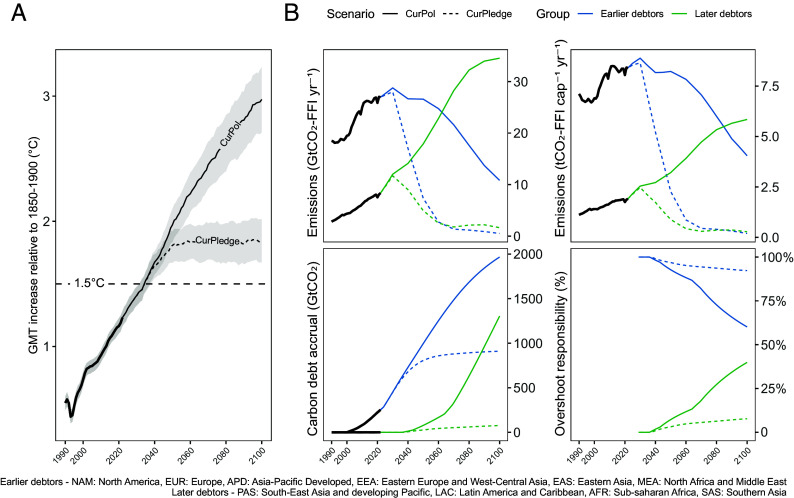
Differentiated carbon debt accrual and overshoot responsibilities under current policies and pledges. (*A*) Estimated median climate overshoot under the assessed scenarios (33rd to 66th GMT anomaly quantiles as ribbon). (*B*) Emissions pathways, carbon debt accrual and corresponding overshoot responsibilities, separating regions that accrue carbon debt by 2030 from those that accrue carbon debt later (see grouping in the caption). This considers an equal cumulative per capita allocation of the total carbon budget from the year 1990, composed of global CO_2_ emissions from fossil fuel and industrial processes (CO_2_-FFI) between the years 1990 and 2023 and an estimated 1.5 °C RCB (50% chance) from the year 2023.

Under our CurPledge scenario (all targets and pledges met), global mean temperature (GMT) is expected to peak at approximately 1.8 °C above preindustrial levels (median) by 2050 (Fig. 3*A*). Under the current policies scenario (CurPol), GMT increase will rise to approximately 3 °C (median) above preindustrial levels by 2100 and continue to rise thereafter. We apply the net-zero carbon debt measure to the regional trajectories underlying these global scenarios (Fig. 3*B*), assigning overshoot responsibility in proportion to accrued debts. Here, we group regions into those that exhaust their allocations before the year 2030 (‘earlier debtors’, approximately 42% of the global population in 2022), and those that do so after 2030 (‘later debtors’, approximately 58% of the global population in 2022). The constituent regional pathways and regional overshoot responsibilities are shown in *SI Appendix*, section 5.

Under the CurPledge scenario, “later debtor” regions initially account for only 3% of total budget exceedance in the year of peak temperature increase (2050), indicating minimal responsibility for the immediate impacts of overshoot in a world where stated climate targets and pledges are met. This relative responsibility increases by the end of the century but remains nevertheless a small share. In contrast, under the CurPol scenario, prolonged late-century emissions cause later debtor regions’ responsibility for budget exceedance to grow rapidly, reaching 40% by the year 2100 and thus contributing to continued temperature rise. In both scenarios, while most regions are expected to accrue some level of carbon debt and associated carbon drawdown responsibilities over the course of the century, near-term responsibilities lie primarily with “earlier debtor” regions that already have or will exhaust their allocations before 2030. These results demonstrate the distinct roles of earlier and later debtor regions in shaping the severity and persistence of global temperature limit exceedance.

**Fig. 4.**
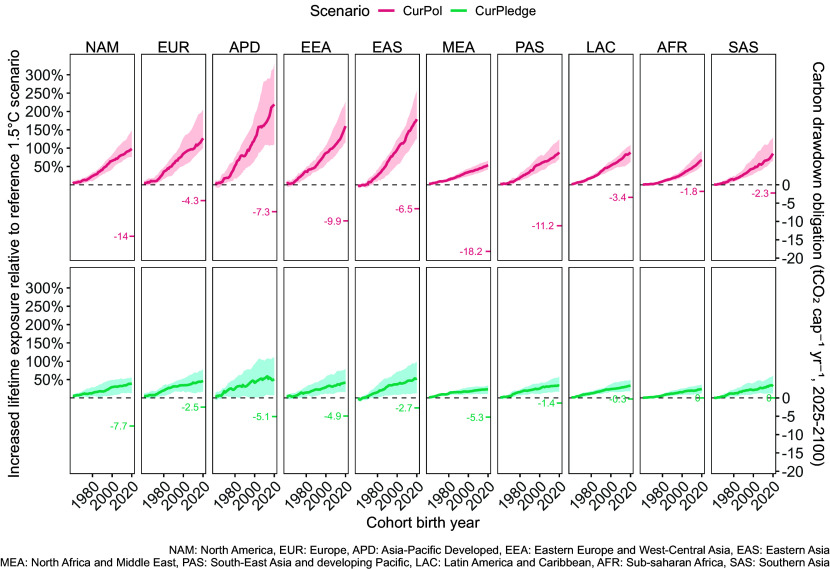
Increased extreme heatwave exposure and carbon drawdown obligations under current policies and pledges. Increase in years of life exposed to extreme (1-in-100-year) heatwaves (primary y-axis) relative to the 1.5 °C reference scenario (IMP-REN, AR6), for all cohorts born between 1960 and 2020 (x-axis). The shaded areas reflect the 33rd and 66th percentile across all global climate model runs and TRQs (*Methods*). The annual equal per capita drawdown obligation to address regional responsibility for scenario overshoot by the year 2100 (starting in 2025) is shown on the secondary y-axis. This refers to an equal cumulative per capita allocation of the total carbon budget from the year 1990, composed of global CO_2_ emissions from fossil fuel and industrial processes (CO_2_-FFI) between the years 1990 and 2023 and an estimated 1.5 °C RCB (50% chance) from the year 2023.

We link these common but differentiated responsibilities for temperature exceedance and corresponding carbon drawdown obligations to realized climate impacts, thereby revealing a dual intergenerational and interregional inequity (Fig. 4). Specifically, we compare equal per capita annual carbon drawdown obligations (assuming completion by 2100) with corresponding increases in lifetime extreme (1-in-100-year) heatwave exposure, illustrating one of the climate impacts resulting from higher temperatures (21, 33). Expressing responsibilities as per capita carbon drawdown obligations enables a like-for-like comparison across regions with vastly different populations and economies. Here, a greater relative per capita carbon drawdown obligation corresponds to a greater relative share of responsibility for budget exceedance. Lifetime extreme heatwave exposures, broken down by age cohorts, are shown relative to a 1.5 °C reference scenario, illustrating the interregional and intergenerational implications of temperature limit exceedance. Because lifetime heatwave exposure under the 1.5 °C reference scenario already varies significantly by region, these increases represent distinct absolute magnitudes across regions (see *SI Appendix*, section 6 for regional reference exposure estimates).

Across regions, the relative changes in exposure and drawdown obligations we quantify illustrate how insufficient climate ambition exacerbates baseline inequities. Under the CurPledge scenario, for instance, zero per capita drawdown obligations in the regions of AFR and SAS nevertheless correspond to similar relative increases in lifetime heatwave exposure as in other regions, notably from higher baselines. Within regions, clear indicators of intergenerational inequity also emerge. In the APD region, for example, the cohort born in 2020 faces an approximate 50% increase (median) in lifetime extreme heatwave exposure under CurPledge—double that of the 1980 cohort. This disparity widens under the CurPol scenario, where the 2020 cohort faces an approximate 220% increase (median), over four times that of the region’s 1980 cohort. At the same time, above-average annual per capita carbon drawdown obligations reflect higher relative regional responsibilities for temperature limit exceedance, reaching −5.1 tCO_2_ capita^−1^ y^−1^ under CurPledge and −7.3 tCO_2_ capita^−1^ y^−1^ under CurPol. Compared with recent regional per capita CO_2_-FFI emissions (9.5 tCO_2_ capita^−1^ in 2022), this implies a rapid shift to net-negative emissions if the region aims to meet its drawdown obligations domestically by 2100. These results highlight the interregional and intergenerational inequities that persist even under optimistic assessments of current global climate ambition. In earlier debtor regions, younger generations are expected to experience more severe climate extremes attributable largely to their regions’ past emissions while also shouldering increasingly large drawdown burdens if obligations are not adequately addressed in the near term, an issue exacerbated by questions of domestic feasibility. In later debtor regions, younger generations must contend with disproportionately large climate impacts despite lower relative responsibility for budget exceedance. These inequities only deepen under our assessment of current policies.

## Discussion

Amid the plurality of perspectives presented under the first Global Stocktake, there has been a shared emphasis on the need to weave equity into a trajectory of increased global climate mitigation ambition (Paragraph 132, 1). Assessments of equity necessary to inform such policy deliberations will need to grapple with a small (and rapidly depleting) RCB consistent with meeting global climate goals (34, 35). In this context, we propose that net-zero carbon debt can be applied as a persistent measure to evaluate regional responsibility for overshoot and quantify drawdown obligations. The formulation of net zero carbon debt we apply here is grounded in principles enshrined in the UNFCCC and the Paris Agreement.

The net-zero carbon debt measure captures responsibility for past emissions, future net-zero ambitions, and resulting overshoot burdens in a single consistent measure. We illustrate a potential application by quantifying regional net zero carbon debt accrual across the scenarios assessed by IPCC WGIII AR6. Through this analysis, we identify sets of regions that consistently do, or do not, accrue net-zero carbon debt in global deep mitigation pathways. We show that these regional net-zero carbon debts can be translated into carbon drawdown obligations to indicate the implications of delayed cuts on future drawdown burdens. Here, we discuss uncertainties associated with comparing gross carbon emissions and net-negative carbon removals, finding an approximate 1:1 long-term temperature equivalence in 1.5 °C-consistent scenarios through experiments conducted with a simple climate model (FaIR). We recognize the need, however, for further work to examine the impacts of coemitted non-CO_2_ greenhouse gas emissions, regional climate impact hysteresis, and the possibility that uncertainties are greater for scenarios at higher warming levels (14, 28–31, 33, 36, 37).

We continue to explore the relevance of this measure for contemporary climate deliberations by modeling two possible global emissions trajectories, one capturing current policies (CurPol), and another capturing stated targets and pledges (CurPledge). When we assess these scenarios through the lens of net-zero carbon debt, important regional differences in expected exceedance responsibilities and drawdown obligations to address this emerge. We differentiate the implications of debt accrual and drawdown between regions with high levels of past emissions (earlier debtors) and those projected to rapidly increase emissions in the future (later debtors). If later debtors could avoid debt accrual expected under the CurPledge scenario, temperature limit exceedance would likely still occur at approximately the same time and reach the same magnitude but would decrease by the end of the century. If early debtors achieve more ambitious pathways in this scenario, temperature limit exceedance would reduce in magnitude and shift out to later in the century when adaptive capacities may have been strengthened. This illustrates the importance of international cooperation to meet global climate goals, as neither group can address the expected exceedance independently.

We then address the question of the domestic relevance of such normative deliberations. To do this, we compare per capita carbon drawdown obligations with projected lifetime exposure to 1-in-100-year extreme heatwaves under the two scenarios, highlighting the interregional and intergenerational inequity implied by low-ambition climate futures. Younger generations in early debtor regions are expected to face significantly higher lifetime heatwave exposure under current policies compared to a reference 1.5 °C scenario. This elevated exposure occurs alongside the potential need for these generations to undertake substantially greater per capita drawdown efforts than the global average, given the disproportionate share of regional responsibility for budget exceedance. In contrast, while younger generations in later debtor regions also face increased exposure, sometimes from a higher baseline, they may only be responsible for a relatively smaller portion of the total drawdown required to address temperature limit exceedance. In both cases, greater climate risks and responsibilities are placed on younger generations. These common but differentiated drawdown obligations and impact comparisons illustrate how the net-zero carbon debt measure can be used to understand and address regressive outcomes of insufficient action that are both regionally mediated and intergenerational in nature.

Our assessment of current global climate ambition can inform the development and evaluation of the next generation of NDCs to be submitted this year. For regions expecting to accrue large net-zero carbon debts, this entails foremost increasing the ambition and specificity of sectoral mitigation plans in subsequent NDCs (12), while quantifying separately targets for permanent carbon dioxide removal technologies to address remaining hard-to-abate emissions (23, 25) and measures to draw down accrued debt. For regions expecting to accrue little to no net-zero carbon debts, this entails careful differentiation between mitigation efforts that bring substantial cobenefits or are cost-competitive, from those that are costly and difficult (38). The latter could be addressed through the structuring of NDC conditionalities that foster necessary technology, capacity, and financial transfers (39, 40).

While our focus here is on mitigation, it is crucial to acknowledge the intertwined issues of loss and damage (41), and the growing need for adaptation funding. Deliberation informed by the net zero carbon debt measure could guide international financial flows for climate-resilient development (2). Mechanisms might also be developed to reconsider international financial debts, where financial debt cancellation, concessional finance, and grants may accelerate near-term adaptation actions in vulnerable regions with little to no overshoot responsibilities.

## Conclusion

Minimizing overshoot and its impacts cost-effectively and equitably requires seeking a politically optimal balance between heightened domestic ambition and internationally supported additional mitigation efforts, informed by considerations of fairness (42). A collapse in global cooperation will likely see increased magnitude and duration of temperature limit exceedance, causing further harm to younger generations in all regions. This is because it is highly unlikely that any single region can unilaterally counterbalance others’ excess emissions under the scenarios we examine, and every additional ton of CO_2_ emitted contributes to global budget exceedance, regardless of fair share claims (19). Norms of global cooperation and perceptions of fairness will therefore be ever more crucial considerations in setting climate ambition and translating this to action in the near term (43), requiring innovative policymaking and international cooperation that maximizes cobenefits (1).

The net-zero carbon debt measure we examine stands out as a useful tool to inform these deliberations for three reasons. First, its normative basis is updateable through international deliberative processes, or judicial interpretation, and can be considered independently by parties to the Paris Agreement in defining their climate mitigation ambition. Second, its robustness is reinforced by growing scientific certainty surrounding estimations of the RCB and trajectories of regional and national emissions, both past and future. Third, it can be used to allocate exceedance responsibility and carbon drawdown obligations following the exhaustion of a RCB, a factor that is crucial considering the pace at which the 1.5 °C budget is being consumed. We argue that a persistent measure of responsibility robust to budget depletion is of value as it draws a line at the acceptable level of warming and guides the return to this level, fairly.

Two futures present themselves. In an uncooperative world, where substantial net-zero carbon debts persist, we will likely witness severe long-term 1.5 °C temperature limit exceedance and associated felt impacts primarily experienced by younger generations. Conversely, in a world where regional net-zero carbon debt is rapidly eliminated through mutually beneficial cooperation, efforts can pivot toward identifying and striving for long-term temperature reduction targets. This shift would mark a transformative step in our collective response to anthropogenic climate change.

## Methods

### The Persistent Accrual of Carbon Debt.

The first step in quantifying net-zero carbon debt is the definition of a total carbon budget, which comprises both total past emissions up to the present year (if responsibility is considered) and the RCB from the present year. In consideration of the long-term temperature goal in the Paris Agreement to hold warming “well-below 2 °C” and to pursue efforts to limit it to 1.5 °C (44), we select a RCB consistent with limiting global warming to 1.5 °C with a 50% likelihood. This budget is estimated to be approximately 247 GtCO_2_ starting from the year 2023 (35). We then define a starting year from which we add past emissions to the RCB as the year 1990 (noting that we also consider an alternative 1850 start year, see *SI Appendix*, section 1). The year 1990 is commonly motivated by the year of the IPCC’s first assessment report when scientific information on climate change started to be communicated systematically to policymakers, though this remains one illustration among several possible choices (3). The total carbon budget thus comprises global CO_2_-FFI emissions from 1990 to 2022 and the estimated 1.5 °C RCB (50% chance) from 2023 (35, 45). In this quantification, we omit CO_2_ emissions from Land Use, Land Use Change and Forestry (LULUCF) and non-CO_2_ greenhouse gases (GHGs), both in the historical and projection periods. We omit CO_2_-LULUCF emissions due to uncertainty in historical estimates (46), different definitions and treatment of land-sector emissions in future scenarios within the Integrated Assessment Model frameworks, and different methods of accounting for LULUCF emissions between National Greenhouse Gas Inventories and scientific models making consistent comparison (Ch. 12, 8, 47, 48). We omit non-CO_2_ GHGs due to the evolving debate regarding the translation of non-CO_2_ climate forces into CO_2_ equivalents for long-term fairness calculations and normative considerations regarding non-CO_2_ GHG emissions floors in the agriculture sector (6, 49–53). Moreover, as we draw on national estimates provided by the Global Carbon Budget project, we exclude emissions attributable to international aviation and shipping, estimated to be 3% of global cumulative CO_2_-FFI emissions from 1990-2022 (46). We recognize that the omission of specific CO_2_ and non-CO_2_ emissions represents a limitation of this work, which we hope will be addressed in future studies. To qualitatively gauge the effect of this omission, we consider the relative regional contributions to warming across emissions source (Fossil, LULUCF) and gas (CO_2_, CH_4_, N_2_O) over the years 1992-2022 (*SI Appendix*, section 2).

We then proceed with budget allocation at the national level. This requires a set of value judgments that align with an interpretation of principles referenced in global treaties and international environmental law (10, 11, 54). In selecting from possible allocation approaches, we start by recognizing the principle of “common but differentiated responsibilities and respective capabilities, in the light of different national circumstances” (CBDR-RC), referenced in the preamble to the UNFCCC and in article 2.2 of the Paris Agreement. We complement this with consideration of the “Polluter Pays” principle, discussed in the climate justice literature (55–58). Polluter Pays in the interpretation used here refers to due consideration of past emissions in future allocations. This principle has been applied successfully in international environmental law in the context of environmental pollution but not yet in the context of climate change, propagating rather through the language of global treaties, e.g., as an interpretation of CBDR-RC (59). Building on these two interpretations, we consider an equal cumulative per capita allocation of the total carbon budget (*SI Appendix*, section 1). National-level budgets are then aggregated to the regional level, following the Integrated Assessment Modeling Consortium 10-region grouping. Our main text thus illustrates the implications of an equal cumulative per capita allocation approach from the year 1990. In our *SI Appendix*, sections 1 and 3, we justify and consider an 1850 starting year and examine allocation approaches that recognize differentiated capabilities.

### The Assessment of Net-Zero Carbon Debt in the IPCC’s AR6 Scenarios Database.

Our first application of the net-zero carbon debt measure is an assessment of all vetted scenarios available in the IPCC WGIII AR6 scenarios database (26, 27). We begin by harmonizing emissions pathways by applying a scaling factor that aligns modeled paths with the historical CO_2_-FFI data in the year 2022 (from ref. 46) and linearly return this scaling factor to 1 by the year 2050, following the approach from IPCC WGIII AR6, but with a later starting year to reflect the most recent data. We freeze harmonized pathways at zero CO_2_-FFI, not allowing net-negative emissions for any of the regions in our assessment. Here, we include a buffer of 100 MtCO_2_ to address pathways that are close to, but do not arrive at net-zero CO_2_-FFI, which is a simplification of the approach applied to all CO_2_ emissions in the IPCC WGIII AR6 (60). We then categorize all regional pathways into decadal net-zero CO_2_-FFI timing groups, such that, for example, pathways achieving net-zero CO_2_-FFI between 2046 and 2055 are grouped into the 2050 net-zero CO_2_-FFI timing group. We combine this set of regional CO_2_-FFI emissions pathways with their historical counterparts (pre-2023) and subtract the resulting cumulative CO_2_-FFI emissions from the regional allocation of the total carbon budget under each allocation approach considered (Fig. 1). This provides a quantification of regional net-zero carbon debts and credits under a given allocation approach for each scenario (see *SI Appendix*, section 4 for further results).

### The Assessment of Net-Zero Carbon Debt under Current Climate Policies and Pledges.

Our second application of the net-zero carbon debt measure is an assessment of two emissions scenarios representing real-world climate policies and pledges. The first reflects a pessimistic current policies scenario (CurPol), which represents no further climate ambition beyond that which is already implemented. The second reflects a very optimistic pledges and net-zero targets scenario (CurPledge), which represents full implementation of pledges contained in both conditional and unconditional NDCs and all net-zero targets. These scenarios capture the range of possible futures, from currently implemented policies through to pledges with little implementation evidence (12). We supplement this assessment with a counterfactual illustrative mitigation scenario from the IPCC WGIII AR6, reflecting a high-renewables narrative (IMP-REN) aligned with the 1.5 °C temperature limit (8). We conduct this work following the framework described by Rogelj et al. (12) with two extensions: First, in an advance on the earlier work, we use vetted scenarios from the IPCC WGIII AR6 scenarios database, rather than the SR1.5 scenarios database. Second, we disaggregate global projections at the ten-region level, rather than the earlier five-region level.

We begin with estimates of GHG emissions to 2030 under scenarios drawing from the 2023 United Nations Environment Program Emissions Gap Report. These include both median estimates and confidence windows, where we focus on the central estimates. We harmonize these estimates and the AR6 database to historical data in the year 2019, using the EDGAR database (61) with regional estimates of emissions where available. Where these are not available, we use global estimates of emissions from the AR6 database (62) in scenario SSP 2_int_lc_50 from the model MESSAGEix-GLOBIOM_GEI 1.0—these represent the most complete set of emissions from the SSP2 MESSAGE family of models. Non-CO_2_ emissions are harmonized to match the historic values until 2019, with a scaling factor (ratio harmonization) that decays to 1 in 2050, whereas both AFOLU and Energy and Industrial CO_2_ emissions are harmonized via an offset that decays over the same time. We take the harmonized data and extend these global emissions pathways using piecewise cubic polynomial interpolation to map the projected Kyoto gas emissions in 2030 onto a carbon price using two ensembles of scenarios, from the MESSAGEix-GLOBIOM and REMIND-MAGPIE models, separately. The interpolation is necessary as global projected emissions do not align perfectly with modeled scenarios. In each case, we use scenarios with names in the format “EN_NPi2020_*00f” (where “*” may be any number and defines a cumulative CO_2 budget over the model period), or “EN_NoPolicy” (a baseline without additional climate action) to construct the relationships between emissions and carbon price, as these provide a sufficient range or fan of scenario emissions and price pathways [as implemented by Rogelj et al. (12)]. We then extend this price by either 2% (current policies) or 3% per year (all pledges and targets). We then map these prices back onto global GHG totals using the aforementioned ensembles of scenarios separately for the CurPol and CurPledge variants. We then disaggregate global GHG totals into regional GHG totals using the time-dependent ratio method, assuming global GHG totals are composed of regional GHG totals that align with time-dependent regional to global emissions ratios in the underlying ensembles of scenarios. Following Rogelj et al. (12), we then calculate the fraction of regional emissions projected to correspond to the country of interest in 2030 based on the net-zero target provided and, for times when the region has positive net emissions, reduce regional emissions by that country’s fraction of regional emissions in that year multiplied by a linear term that ramps up from zero in 2030 to one in the net zero year. The EU is considered a single country for the purposes of this calculation. Net-zero targets were taken from the Net-Zero Tracker (https://zerotracker.net/) and the Climate Action Tracker (https://climateactiontracker.org/). These Kyoto basket emissions are broken down into components using data from the same scenario ensembles using the tool Silicone (63), following the same approach as used by Rogelj et al. (12), except on a regional basis for all emissions except the F-gases (where data are not always available at a regional level). The complete set of emissions is then run-through the simple climate model FaIR v2.2, using a calibration (calibration v1.4.1; 32) that is constrained upon observed climate change from 1850 to 2022 and assessed ranges of key climate indicators (e.g., climate sensitivity) from the IPCC Sixth Assessment Report Working Group I (64). We do this to enable heatwave exposure assessment, described in the subsequent section. To enable comparison with more recent historical datasets, we subsequently harmonize regional CO_2_-FFI pathways once more to CO_2_-FFI data in the year 2022 (from ref. 46) and linearly return this scaling factor to 1 by the year 2030, because actual emissions deviate from modeled emissions between the years 2019 and 2022. This harmonization is a pragmatic decision to align modeled paths with recent historical counterparts. The resulting difference in projected cumulative emissions is less than 2.5% in most regions. Finally, to calculate regional net-zero carbon debt, we sum cumulative CO_2_-FFI emissions to the year of regional net-zero CO_2_-FFI and subtract this from regional allocations as before. This quantification is then used to determine the relative regional responsibility for overshoot. Further details are provided in *SI Appendix*, section 5.

### The Assessment of Additional Lifetime Extreme Heatwave Exposure.

We extend the assessment of our two scenarios with an evaluation of corresponding lifetime additional extreme heatwave exposure using the approach originally laid out by Thiery et al. (20). Lifetime extreme heatwave exposure indicates the cumulative number of years with at least one extreme heatwave that an individual born in a given region and given year would experience over their expected lifetime under a given GMT pathway. Extreme heatwaves are defined as cases where the HeatWave Magnitude Index daily (HWMId) (65, 66) of a given year exceeds the 99th percentile of the HWMId distribution under preindustrial climate conditions of that grid cell (67). In our case, this represents a “1-in-100” year likelihood extreme heatwave. Annual extreme heatwave occurrence is computed from the results of four global climate models (GCMs) contributing to the Coupled Model Intercomparison Project phase 5 (CMIP5) that were bias-adjusted as part of the Inter-Sectoral Impact Model Intercomparison Project phase 2b (68) and remapped to the scenarios considered here using 30-y running mean GMT anomalies from the respective CMIP5 models (see replication archive for model and run details). This results in our case in 4 GCM × 3 representative concentration pathway (RCP) runs, which equals in total 12 GCM-RCP runs from which we sample and remap to the assessed scenarios. Here, for each GCM-RCP run, we identify the GMT anomaly closest to the anomaly in our pathway, which may be in a different year, and map this to our pathway if the difference remains below 0.2 °C. We compute the lifetime extreme heatwave exposure by birth cohort under an illustrative 1.5 °C scenario (AR6 IMP-REN) and under the two scenarios (CurPol and CurPledge). This is done for 13 temperature response quantiles (TRQs) for each emissions scenario. That is, for each cohort we generate 13 estimates of lifetime exposure for all of the 12 GCM-RCP runs that map to the expected scenario temperature pathways. The two emissions scenarios are then compared against the illustrative 1.5 °C scenario within each GCM-RCP and TRQ pathway, resulting in a consistent within GCM-RCP-TRQ lifetime exposure comparison for each age cohort. We conduct this comparison both in terms of anomalies which reflect the absolute differences in lifetime years with extreme heatwave exposure and exposure multiplication factors (EMFs) which reflect the relative factor change in lifetime years with extreme heatwave exposure. We report the median anomalies and EMFs using all GCM-RCP-TRQ combinations for each cohort and include underlying model and temperature response uncertainty by describing the 33 to 66% quantile range. The anomalies and EMFs thus calculated describe the probabilistic increase in lifetime extreme heatwave exposure under a given scenario and relative to the illustrative 1.5 °C scenario. It is important to note here that regional differences in exposure under the illustrative 1.5 °C scenario are must be considered, which we discuss in the main text and illustrate in *SI Appendix*, section 6.

## Supplementary Material

Appendix 01 (PDF)

## Data Availability

A replication archive and all necessary data can be found at https://doi.org/10.5281/zenodo.14915595 (69).

## Footnotes

^*^Note that a subset of countries within a region may remain in credit while the region in aggregate accrues debt.
