# Supplementary material for: Using net-zero carbon debt to track climate overshoot responsibility
Source: Proc Natl Acad Sci U S A. 2025 Mar 24;122(13):e2409316122. doi: 10.1073/pnas.2409316122 (PMC12002226; doi:10.1073/pnas.2409316122)
Supplement: Supplementary file 1 — Appendix 01 (PDF) [file pnas.2409316122.sapp.pdf]

Using net-zero carbon debt to track climate overshoot responsibility

Setu Pelz*[1], Gaurav Ganti[1,2,3,4], Robin Lamboll[5], Luke Grant[6], Chris Smith[1,6], Shonali Pachauri[1], Joeri Rogelj[1,5], Keywan Riahi[1], Wim Thiery[6], Matthew J. Gidden[1,4]

[1] International Institute for Applied Systems Analysis, Laxenburg, Austria
[2] Potsdam Institute for Climate Impact Research, Member of the Leibnitz Association, Potsdam, Germany
[3] Geography Department and IRI THESys, Humboldt-Universität zu Berlin, Berlin, Germany
[4] Climate Analytics, Berlin, Germany
[5] Centre for Environmental Policy and Grantham Institute for Climate Change and the Environment, Imperial College London, London, UK
[6] Department of Water and Climate, Vrije Universiteit Brussel, Brussels, Belgium.

*Corresponding author, pelz@iiasa.ac.at

Keywords: Overshoot, Carbon debt, Intergenerational equity, Interregional equity, Impacts

Replication archive DOI: 10.5281/zenodo.14915596

Supporting Information

Contents

1. Carbon budget allocation approaches considered 2
2. Comparing relative contributions to warming across components and gasses 4
3. Illustrating fair share quantifications of the total carbon budget from 1990-2020 6
4. Assessing CO2-FFI emissions pathways from the IPCC WGIII AR6 scenarios database 7
5. Assessing CO_2-FFI emissions pathways under current policies and pledges 10
6. Uncertainties in heatwave exposure estimation under current policies and pledges ... 15
7. On the temperature equivalence of carbon emissions to carbon removals 17

1. Carbon budget allocation approaches considered

Equation (1) describes our main carbon budget allocation approach in mathematical terms, where *y* indicates the starting year of allocation (1990), *p* indicates the year from which the remaining carbon budget is determined (2023), *z* indicates the final year of allocation (2050), *c* designates the study country of total countries *n*, and *p-1* denotes the year before the remaining carbon budget is calculated (2022). Considering an earlier allocation year simply requires modifying the starting year of allocation (*y)* back, e.g. to the year 1850. The year 2050 is selected as the final year of allocation, after which allocations are held fixed. This is generally in line with the year of global net-zero CO_2 with no or limited overshoot of 1.5°C across pathways assessed by the IPCC (IPCC, Table SPM 2 2022).

$$RemainingBudget_{p,z,c} = \frac{CumulativePopulation_{y,z,c}}{\sum_c^n (CumulativePopulation_{y,z,c})} \times GlobalBudget_{y,z} - CO2_{y,p-1,c} \quad (1)$$

We then consider an alternative that adjusts these equal cumulative per capita allocations in inverse proportion to the 'Ability to pay', with the inverse transformation function having important implications for the resulting aggregate (see Figures S2a & S2b). 'Ability to pay' in the interpretation used here refers to relative budget adjustment in inverse proportion to the ability to invest in mitigation efforts. This principle has roots in climate justice literature and philosophy and is similarly found in the language of global treaties, e.g., in reference to the necessary financial support from developed to developing countries and as another interpretation of CBDR-RC. We quantify this using the proxy of gross domestic product (GDP) per capita both in purchasing power parity (PPP) terms and in terms of market exchange rates (MER), measured from the year 1990.

Equation (2) describes the adjustments for ability to pay, scaling per capita remaining allocations in inverse proportion to the relative cumulative per capita GDP from 1990-present. $\widehat{x_{y,p-1,c}}$ reflects an inverse transformation of the cumulative GDP over cumulative capita for country *c* from the starting year of allocation *y* (1990) to the year before which modelled remaining carbon budgets are defined *p-1* (2022). In contrast to other studies using the per capita GDP indicator, we consider cumulative GDP over cumulative capita from the first year of allocation up to the year p-1 (2022), to update capabilities changing over this period. Here, when considering historical responsibility prior to 1990, we allocate historical budgets in

analogue to Equation (1), setting the starting year of allocation (*y*) to e.g. 1850, and the final year of allocation (*z*) to 1989, and subtracting known CO2-FFI emissions over the same period. We then add to this the allocation over the period 1990-2050 using Equation (2).

$$RemainingBudget_{p,z,c} = \frac{\widehat{x_{y,p-1,c}} \times CumulativePopulation}{\sum_c^n \left(\widehat{x_{y,p-1,c}} \times CumulativePopulation_{y,z,c}\right)} \times GlobalBudget_{y,z} - CO2_{y,p-1,c} \quad (2)$$

In scaling for ability to pay, a set of substitutable inverse transformations can be applied as noted below, each reflecting a distinct value judgement on the desired relationship between the range of the original indicator value and the corresponding inverse range. We suggest F(x) = 1/sqrt(x) in this work, in contrast to other possible choices, such as the more severe (e.g. F(x) = 1/x) and more the lenient (e.g. F(x) = 1/asinh(x)).

2. Comparing relative contributions to warming across components and gasses

Here we consider the implications of our focus on CO_2 emissions from energy and industrial processes (CO_2-FFI). We have noted the reasoning for this decision in our Methods section. The figure and work shown here is thus intended to provide a qualitative assessment of the implications of this decision and its use in the allocation of a remaining carbon budget (Figure S1). Here we consider the period 1992-2022, which reflects the union of the time period considered in our main specification (equal cumulative per capita allocation from the year 1990) and the historical data made available by Jones et al. (1).

The data provided by Jones et al. (1) includes estimates of the contribution to global warming from the year 1992 to the year 2022 across a range of gasses (CO_2, CH_4, N_2O) and components (Fossil, LULUCF). We use this data to compute an allocation analogous to that which we use in our work, namely an equal cumulative per capita allocation, in this case, of the total anthropogenic warming over the period 1992-2022. This allows us to consider how the selection different gasses and components may influence relative allocations. We first determine the total warming over the period over the period by gas and component and then allocate this in an equal cumulative per capita manner to each country. We subtract the actual warming contribution through each gas and component by each country from this allocation. The difference is presented at the aggregated regional level difference where values above 0 indicate contributions to warming beyond the allocation, and values below 0 indicate contribution to warming below the allocation.

We see that the CO_2-FFI is the dominant driver of warming inequality across the gasses and components (Figure SI 1a), and that the inclusion of CO_2-LULUCF to CO_2-FFI (Figure SI 1b and 1c) would result e.g. in greater allocations to SAS, and lower allocations in AFR, LAC and PAS. We also observe that the consideration of all GHGs changes relative contributions. As noted in our methods section, these alternatives require the consideration of a series of unresolved equity issues, such as the temporal differentiation of deforestation between developed and developing countries and the issue of agricultural emissions and their non-equivalence with fossil fuel emissions. Future work should consider how to address non-CO_2 and LULUCF emissions, which will require context-specific considerations. See Li et al. (2) for recent discussion on this topic.

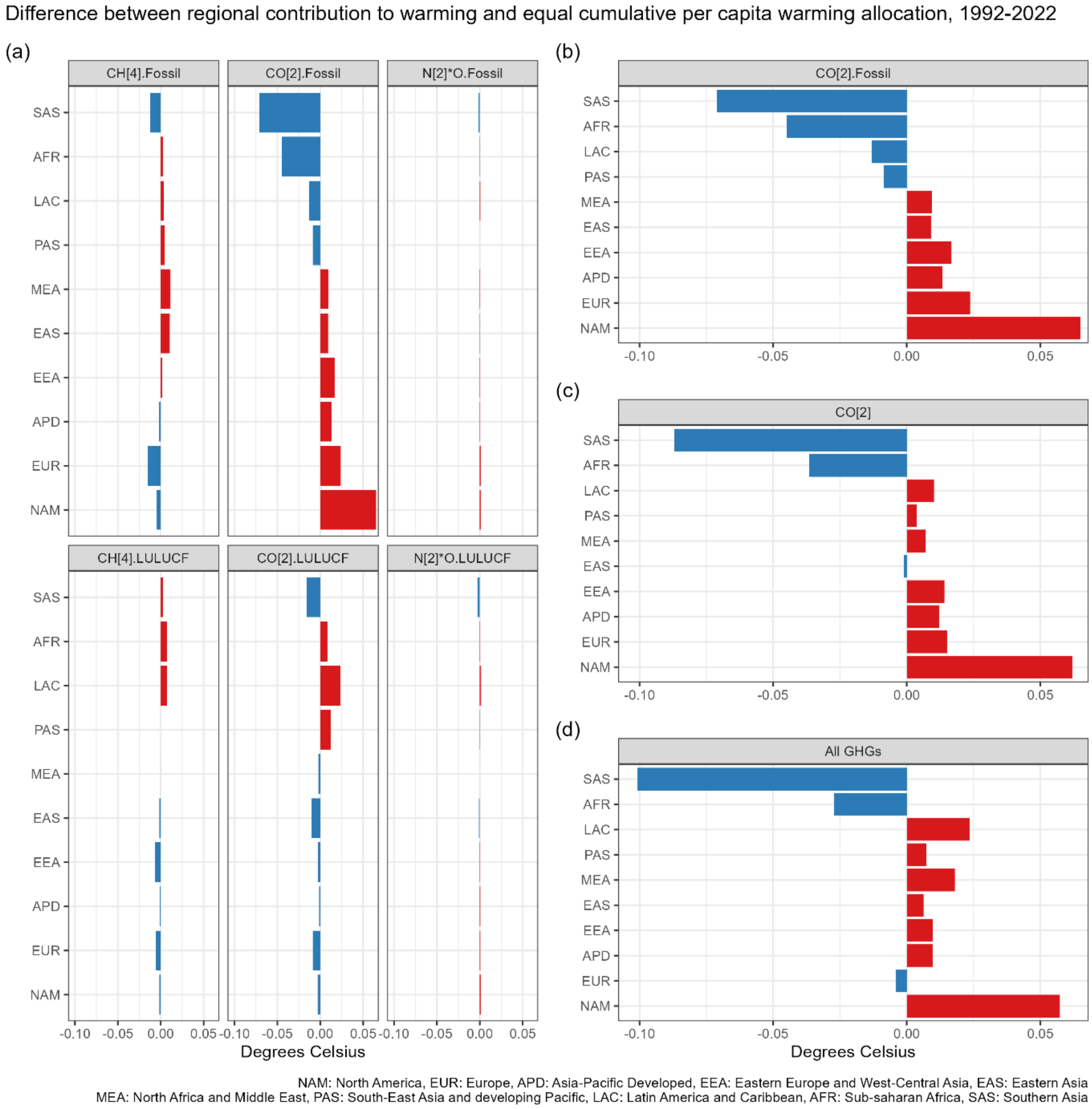

Figure S1 – Comparing relative contribution to global warming between from the period 1992-2022 across distinct gasses and components. This figure illustrates the relative contributions to global warming across different gases and components from 1992 to 2022. Each region's contributions are calculated by first determining the total warming by gas and component, then allocating this amount equally on a cumulative per capita basis. The figure shows the deviation of actual contributions from these allocations, where values above zero represent contributions exceeding the allocation, and values below zero signify contributions below the allocated level.

3. Illustrating fair share quantifications of the total carbon budget from 1990-2020

Figures S2a and S2b illustrate fair share quantifications across a range of possible allocation approaches and timeframes, shown both in absolute and per capita terms.

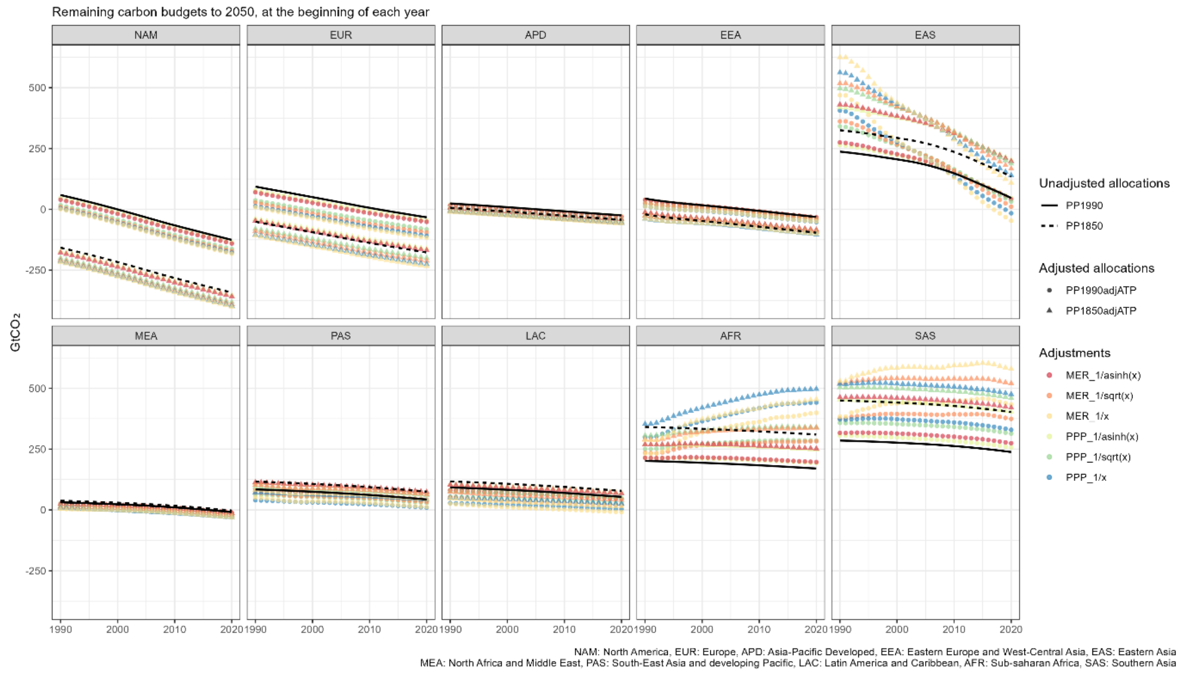

Figure S2a - Regional absolute allocations of the total carbon budget at the beginning of each year from the years 1990-2020, across allocation approaches consistent with the value judgements described in the Methods.

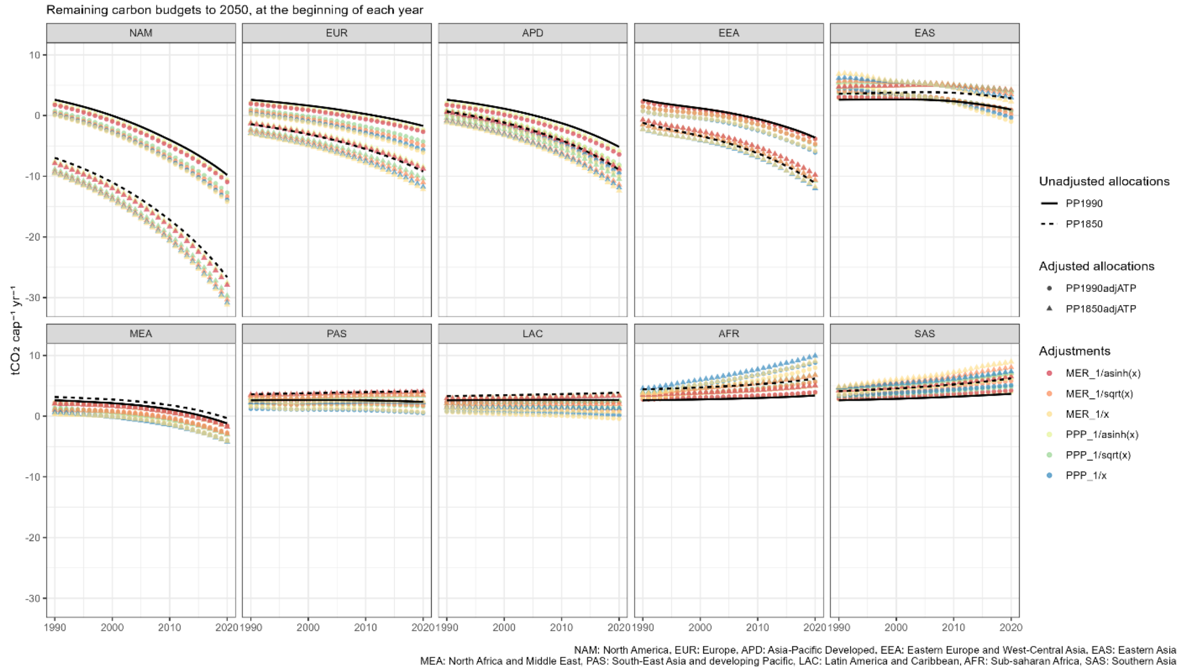

Figure S2b - Regional remaining per capita (to 2050) allocations of the total carbon budget at the beginning of each year from the years 1990-2020, across allocation approaches consistent with the value judgements described in the Methods.

4. Assessing CO2-FFI emissions pathways from the IPCC WGIII AR6 scenarios database

Figure S3 visualises the scenario harmonisation effect on cumulative CO_2-FFI emissions.

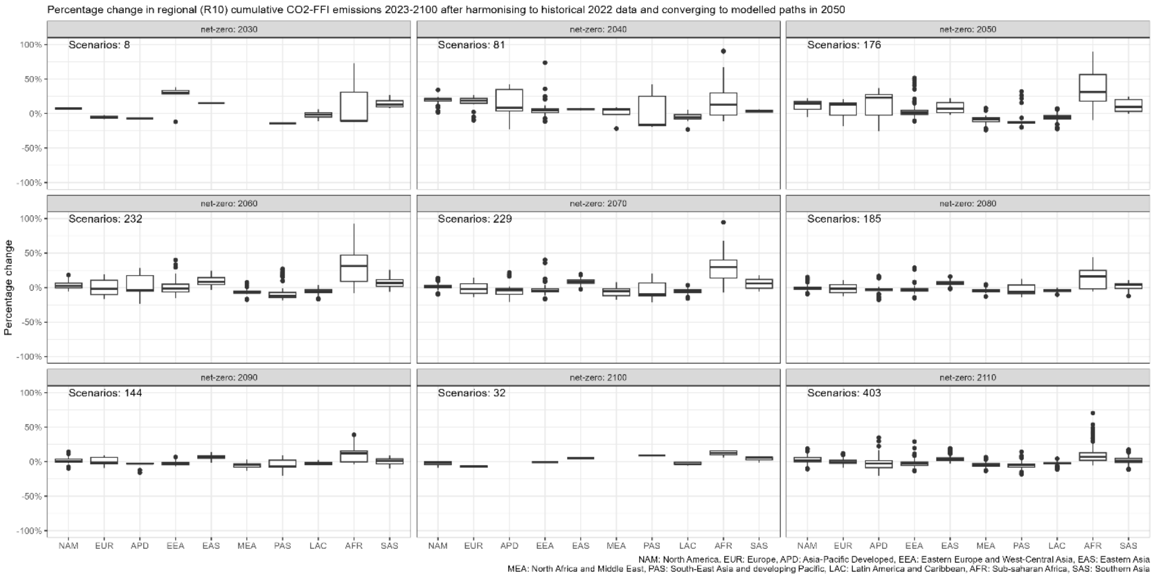

Figure S3 - The effect of harmonizing modelled CO_2-FFI emissions across regional pathways derived from the IPCC WGIII AR6 scenarios database to historical data in the year 2022 on cumulative projected emissions from 2023-2100. 432 scenarios were considered in our analysis, reflecting the subset with regional net-zero CO_2-FFI years not exceeding the 2090-year bin.

Figures S4a shows that the regional grouping discussed in the main text is consistent across other allocation approaches considered, with the exception of EAS under an 1850 start year.

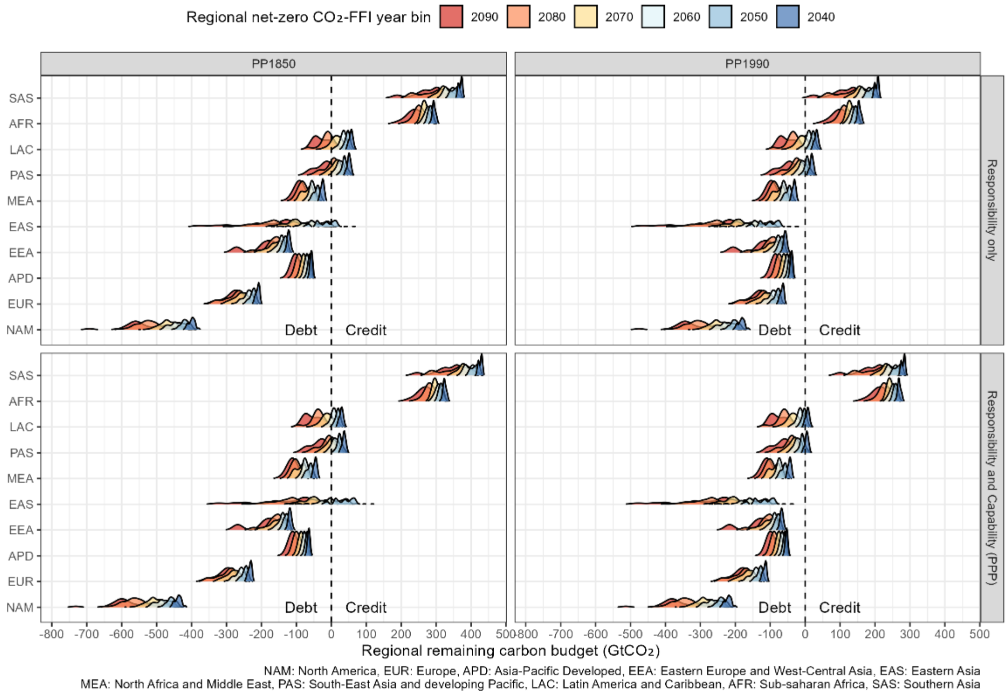

Figure S4a – Assessing net-zero carbon debt across two allocation periods and considering both unadjusted equal cumulative per capita allocations and allocations adjusted to address ability to pay. The latter is shown using purchasing power parity (PPP) and applies the 1/sqrt(x) inverse transformation value judgement.

Figure S4b compares per capita drawdown obligations and overshoot responsibilities by net-zero timing across regions. Notably, this reveals similar per capita drawdown obligations under late net-zero targets for both APD and NAM, despite the latter region having a much larger absolute carbon debt. This figure also reveals differing carbon drawdown and overshoot responsibility dynamics between regions with greater past emissions and those with more recent emissions growth. For example, NAM is projected to be responsible for at least one fifth of overshoot in nearly all scenarios and relative overshoot responsibility is evidently inversely proportional to drawdown obligation for this region. This is because in the AR6 WGIII scenarios where NAM achieves net-zero early, global net-zero also typically occurs earlier as the rest of the world mitigates strongly. Thus, NAM's past emissions play a stronger role in terms of overshoot responsibility while overall exceedance reduces. The opposite dynamic is evident for EAS, which has lower relative past emissions and a large population. Here, while global net-zero similarly occurs earlier in AR6 WGIII scenarios where EAS achieves earlier regional net-zero, EAS's relatively low past responsibility implies that both overshoot responsibility and drawdown obligations reduce in parallel.

Figure S4c illustrates the implications of different drawdown timeframes, we quantify annual carbon drawdown obligations necessary to address net-zero carbon debts for two regions, North America (NAM) and Eastern Asia (EAS), under median pathways reaching net-zero CO_2-FFI by 2050 or 2070 (Figure 2b, main text). In both scenarios, we assume carbon drawdown begins pre-emptively in 2025 and ends by either by 2050 or 2100, distributing carbon drawdown obligations on an equal per capita basis over these timeframes. Completing drawdown by 2050 minimises responsibility for climate overshoot, but requires immense drawdown effort in the near term, starting at -7.4 $GtCO_2\ yr^{-1}$ in NAM and -4.1 $GtCO_2\ yr^{-1}$ in EAS under the median 2050 net-zero pathway. This rises under the median 2070 regional net-zero pathway, increasing particularly quickly for EAS and pointing to important differences in past and future carbon debt accrual across regions (see Figure S4b). Spreading this effort to the year 2100 lowers near-term obligations but contributes to higher and longer overshoot, as greater drawdown occurs in the latter half of the century.

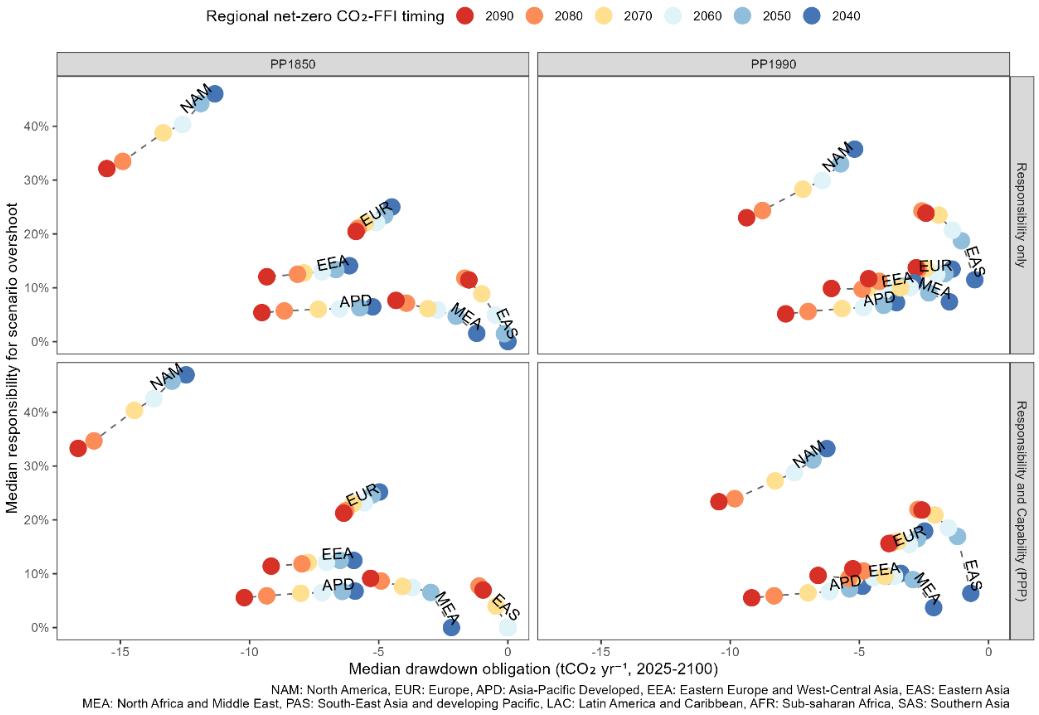

Figure S4b - Comparing median per capita drawdown obligation, assuming this occurs between 2025-2100, against the relative responsibility for overshoot in the corresponding scenario, considering both unadjusted equal cumulative per capita allocations and allocations adjusted to address ability to pay. The latter is shown using purchasing power parity (PPP) and applies the 1/sqrt(x) inverse transformation value judgement.

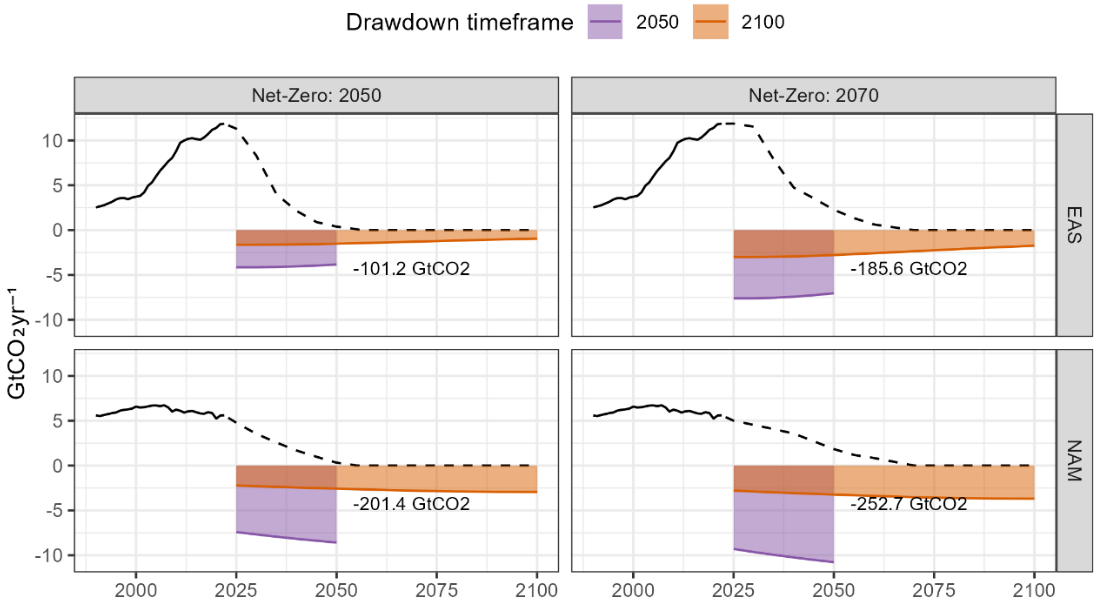

Figure S4c - Future median emissions trajectories (dashed line) to net-zero in 2050 or 2070 for the regions North America (NAM) and Eastern Asia (EAS), shown alongside the carbon drawdown obligations (ribbon) they imply. Carbon drawdown obligations are spread equally per capita from the year 2025 to either 2050 or 2100, demonstrating how drawdown timeframes affect effort distributions. Here we consider the allocation approach presented in the main text.

5. Assessing CO_2-FFI emissions pathways under current policies and pledges

Figure S5 visualises the coverage of net-zero targets across all analysis countries we group into regions and represent here in terms of relevant indicator aggregates. This shows for example that regions with greater shares of creditors (as of 2019, under the approach considered in our main text) typically did not have net-zero targets in legislation.

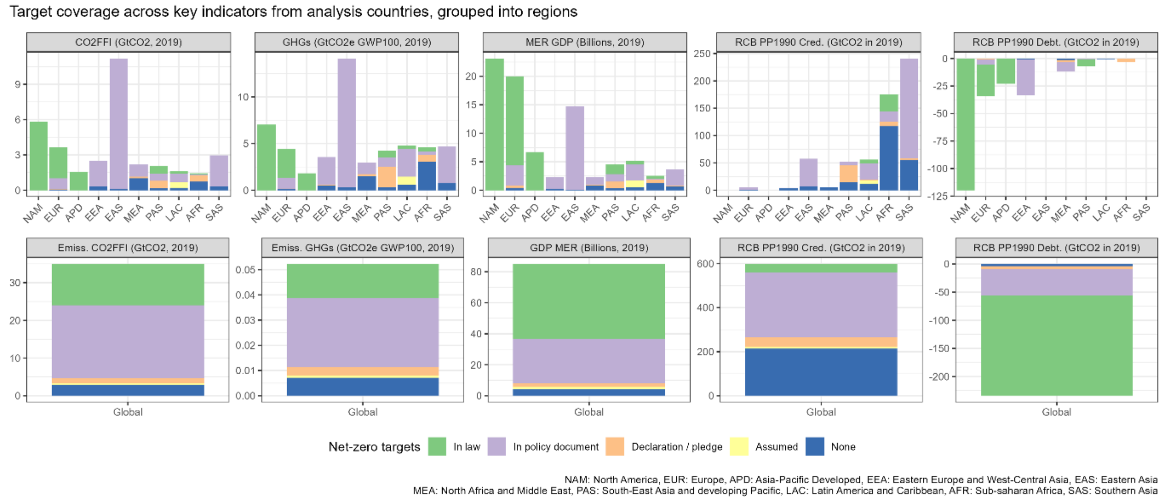

Figure S5 – Coverage of net-zero targets across study countries, aggregating key indicators into regional groups.

Figure S6 visualises the scenario harmonisation effect on cumulative CO_2-FFI emissions.

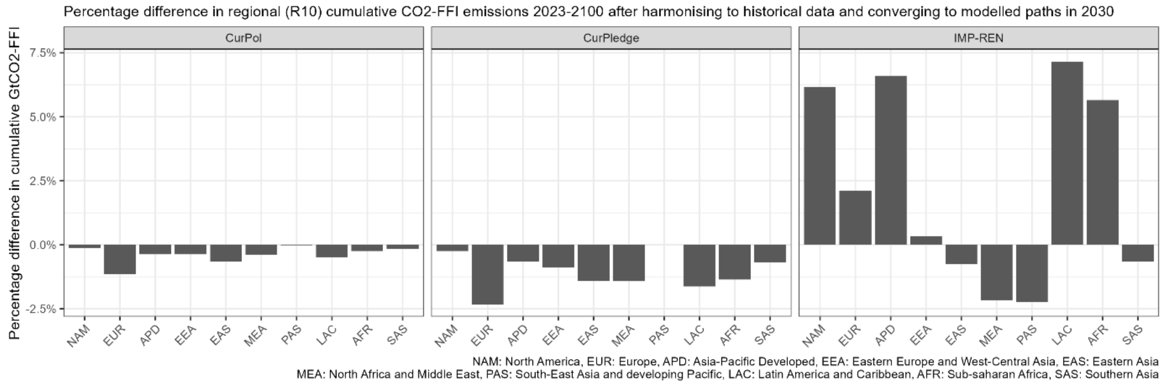

Figure S6 - The effect of harmonizing modelled assessed CO_2-FFI emissions to historical data in the year 2022 and converging in the year 2030 on cumulative projected emissions from 2023-2100. We also show the result of harmonizing the illustrative IMP-REN pathway to historical data. Here, as we do not require quantification of associated net-zero carbon debts and alignment with recent data, we harmonize to the year 2015 and converge in 2050 as per IPCC WGIII AR6.

Figure S7a plots the modelled harmonized regional CO_2-FFI emissions pathways, their global aggregate and the resulting global mean temperature increase relative to the period 1850-1900. Here we also show results for the illustrative 1.5°C scenario (IMP-REN). Figure 7b

visualizes the illustrative consumption of allocated remaining carbon budgets corresponding to these emissions paths from 1990-2100 under our main allocation approach.

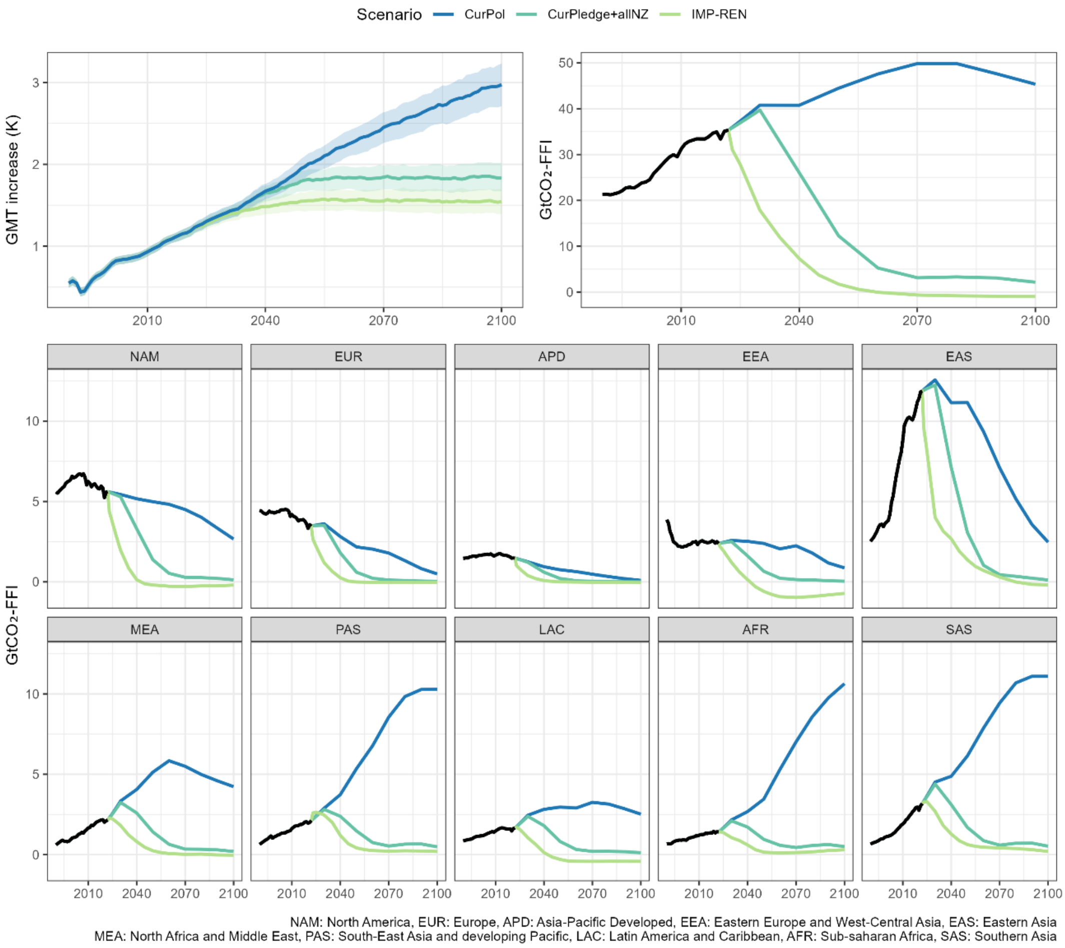

Figure S3a - Modelled harmonized regional CO_2-FFI emissions pathways under the two new scenarios, compared with the IPCC AR6 WGIII IMP-REN 1.5°C low overshoot pathway.

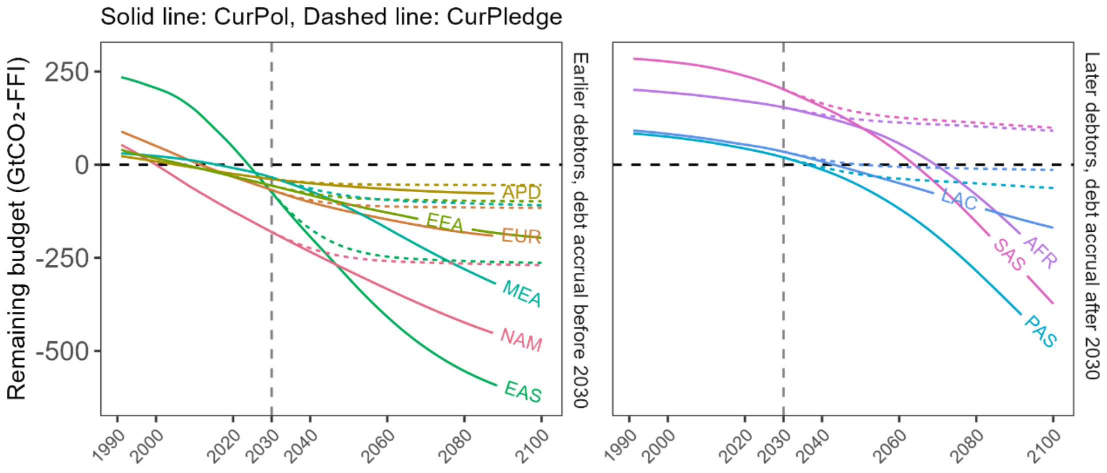

Figure S7b – The consumption of allocated remaining carbon budgets from the year 1990-2100 under our main allocation approach, visualizing the differences in debt accrual between 'earlier debtors' (left) and 'later debtors (right), for the two scenarios we consider.

Figures S8a illustrates the change in CurPledge and CurPol scenario regional overshoot responsibilities under all allocation approaches considered in this work. Figure S8b illustrates how responsibilities shift between CurPledge and CurPol under our main allocation approach.

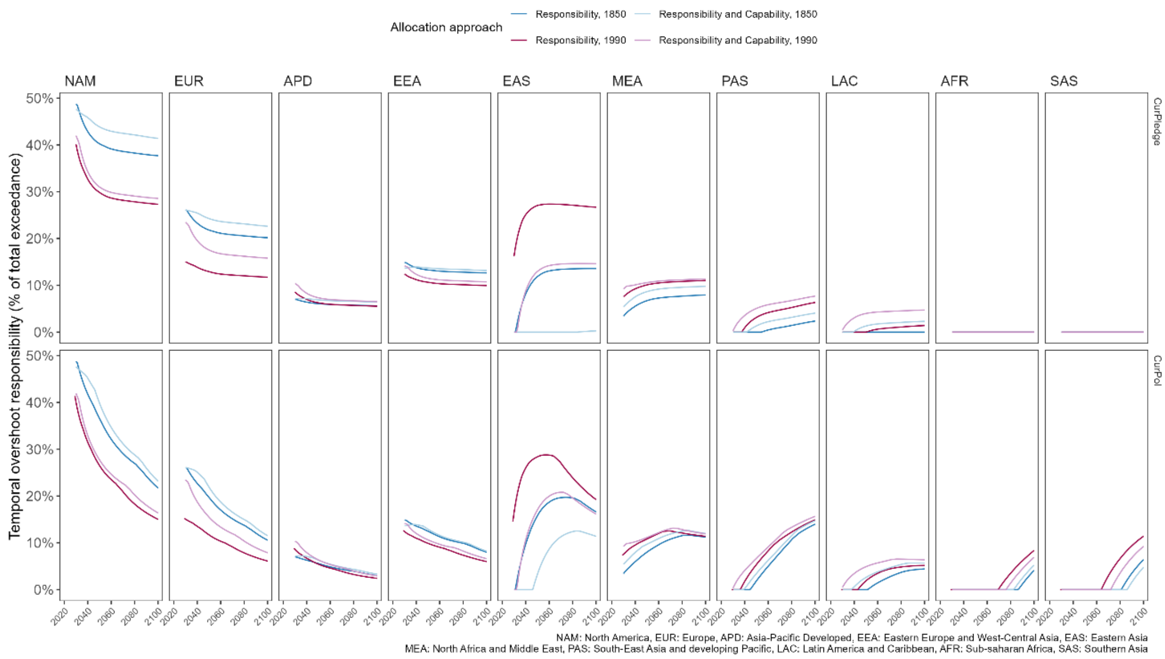

Figure S8a – Relative regional 1.5°C RCB exceedance responsibility from 1990-2100 across selected allocation approaches, under the two new scenarios. Adjustments for 'Ability to pay' using the proportional inverse scaling of cumulative GDP per cumulative capita described in the Methods and SI are shown using purchasing power parity (PPP) conversions. The unadjusted PP1990 allocations reflect the quantifications discussed in the main analysis.

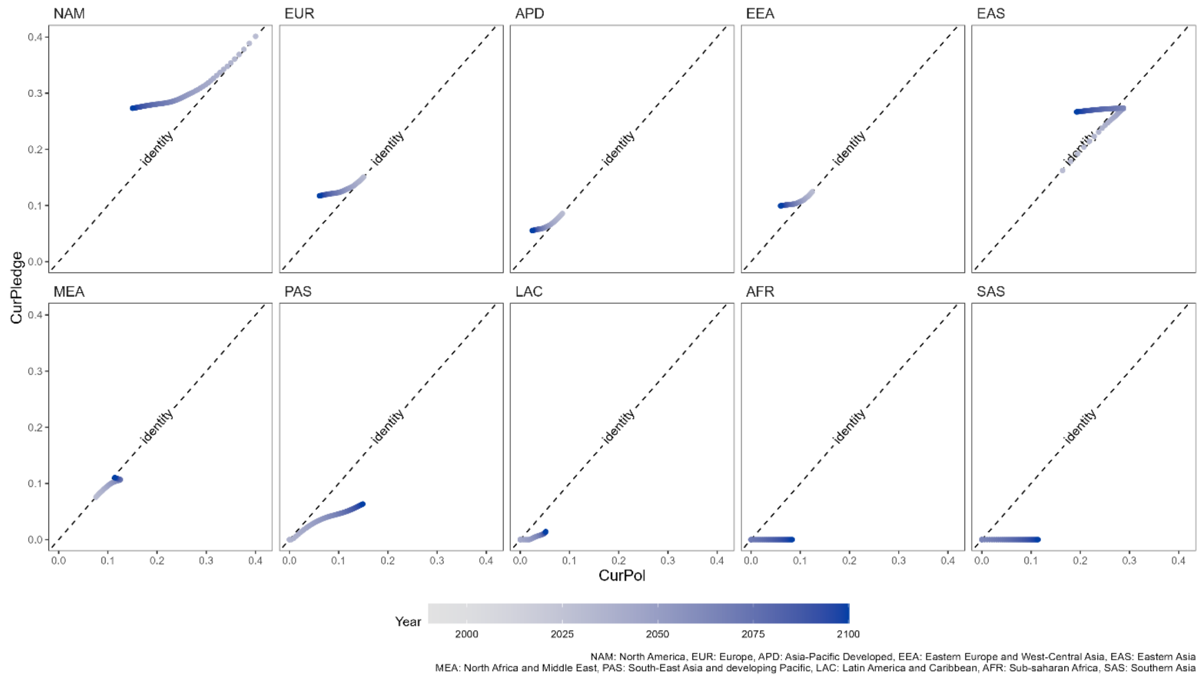

SI Figure 8b – Comparing relative regional overshoot responsibilities over time between CuPledge and CurPol under our main allocation approach.

Figure S9a describes the regional emissions pathways corresponding to the groups shown in Figure 3 in our main text. Here, we also illustrate annual carbon drawdown obligations (under an equal cumulative per capita allocation, from the year 1990) to address carbon debts accrued by the year 2100. We assume drawdown begins pre-emptively in the year 2025 and is spread in an equal per capita manner over the course of the century. These illustrative equal per capita drawdown obligations may once again be addressed through additional mitigation efforts (that would reduce drawdown necessary) and permanent carbon dioxide removal, noting our consideration of the uncertainties in the long-term warming equivalence of gross emissions to net-negative removals. For some earlier debtor regions, even under the optimistic CurPledge scenarios, we find that populations may be assigned substantial full century regional carbon drawdown obligations from this year. For later debtor regions, absolute drawdown obligations are far lower (and relatively lower still, in per capita terms). Nevertheless, under both scenarios, all regions are expected to address some level of carbon debt accrual over the course of the century, notwithstanding that this debt accrues at different times and to significantly different degrees.

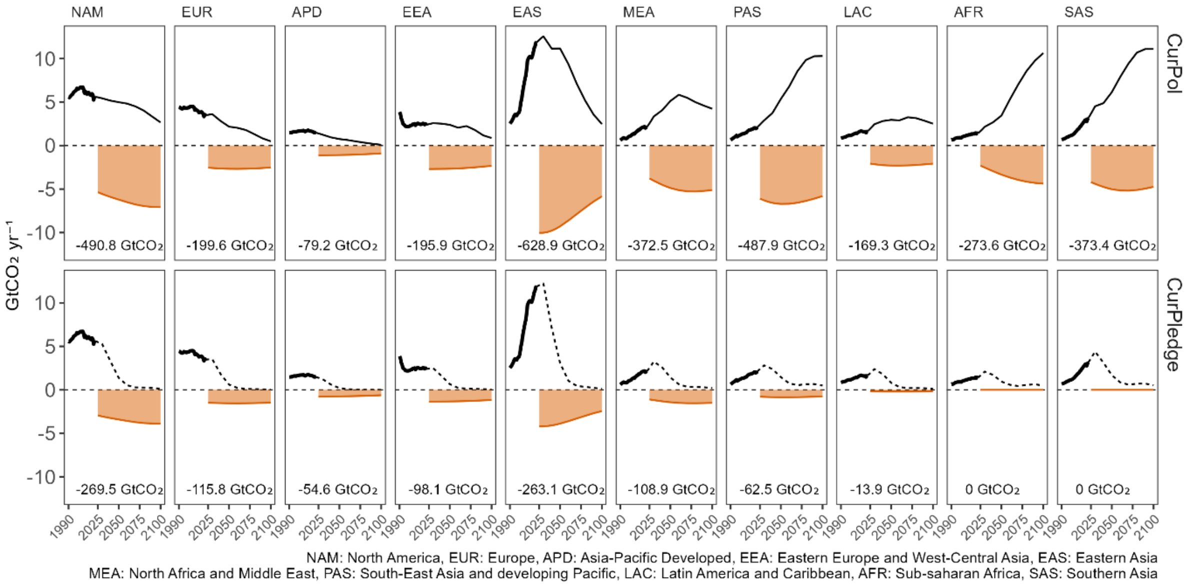

Figure S9a – Future emissions trajectories (dashed lines) for each scenario are shown alongside the carbon drawdown obligations (negative ribbons) they imply. The drawdown obligations implied by these illustrative scenarios are spread equally per capita from the year 2025 to the year 2100. This considers an equal cumulative per capita allocation of the total carbon budget from the year 1990, comprised of global CO_2 emissions from fossil fuel and industrial processes (CO_2-FFI) between the years 1990-2023 and an estimated 1.5°C RCB (50% chance) from the year 2023.

Figure S9b compares global and regional per capita carbon drawdown obligations under CurPledge and CurPol, assuming that drawdown is completed over the period 2025-2100 as in Figure S9a. This illustrates how regional drawdown obligations vary from the global population-weighted average, providing an indication of relative overshoot responsibilities.

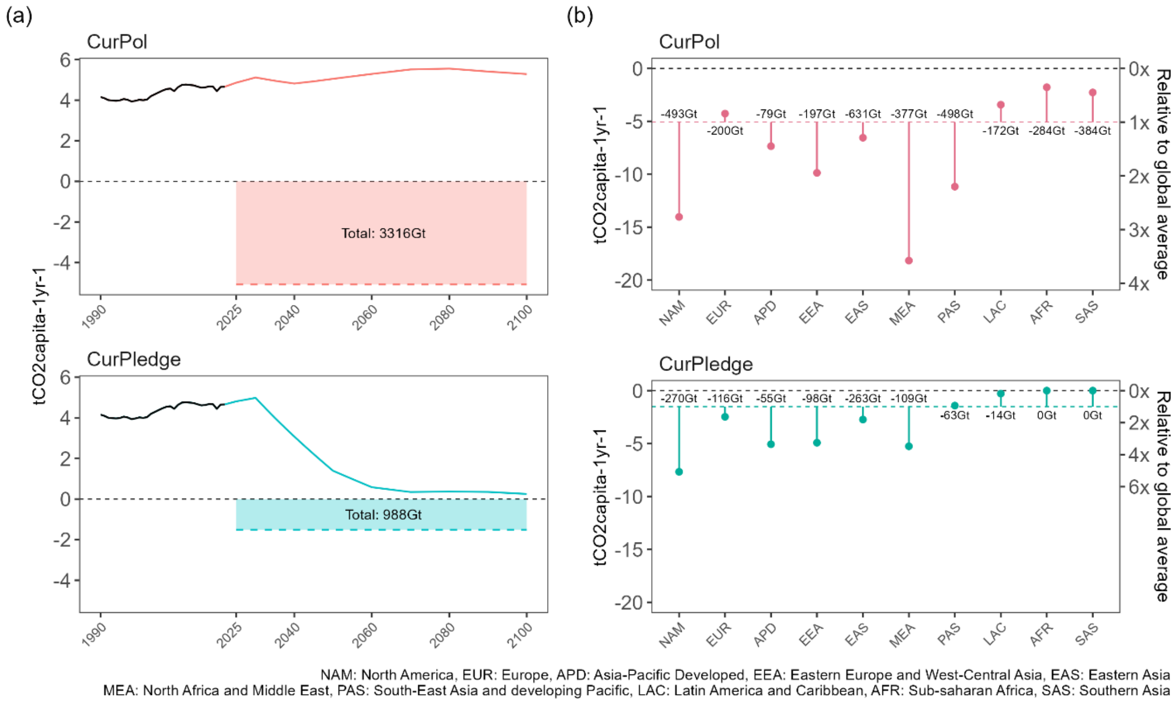

Figure S9b – *Regionally differentiated drawdown obligations under current policies and pledges.* ***(a)*** *Global per capita CO2 emissions from fossil fuel and industrial processes (CO2-FFI) between the years 1990 and 2100 under current policies (CurPol) and pledges (CurPledge). The associated average global per capita carbon drawdown required to return to 1.5°C over the period 2025-2100 is shown by the dashed line.* ***(b)*** *The regional per capita drawdown obligation (2025-2100) under an equal cumulative per capita allocation of the total carbon budget from the year 1990.*

6. Uncertainties in heatwave exposure estimation under current policies and pledges

Figures S10a and S10b visualise uncertainties in heatwave exposure estimates, for the age cohort born in the year 2020.

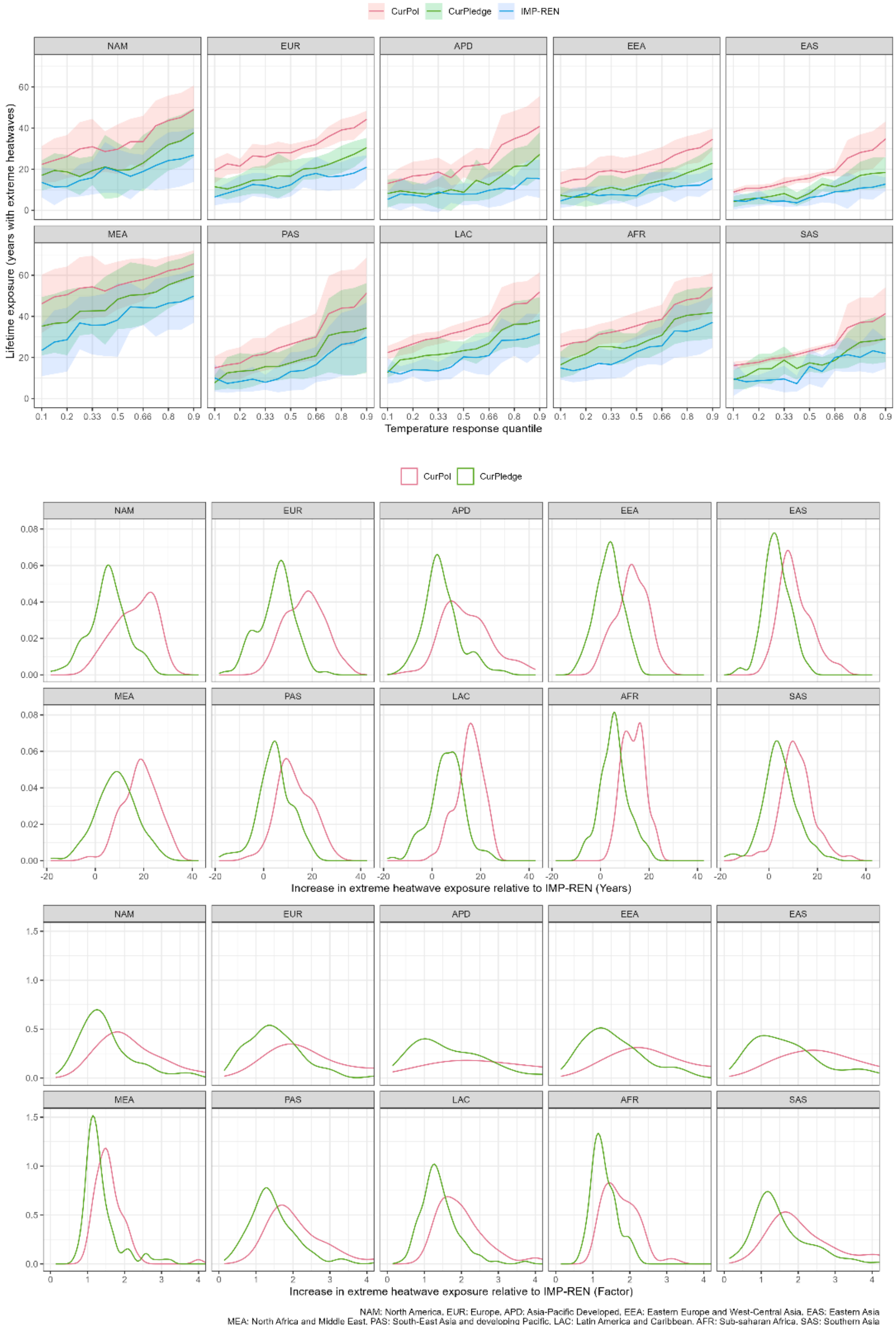

*Figure S4 – **(a)** Median (line) lifetime years of exposure to extreme heatwaves across all temperature response percentiles, for the cohort born in 2020. Ribbons describe the 33rd and 66th quantiles of exposure estimates across all GCM x run x temperature response quantile combinations. **(b)** Density plots describing the increase in lifetime years with extreme heatwaves relative to IMP-REN and corresponding exposure multiplication factor relative to IMP-REN, for all GCM x run x temperature response quantile combinations, for the cohort born in 2020.*

Figure S11 describes regional differences and uncertainties in baseline (1.5°C reference) and scenario (CurPledge, CurPol) extreme heatwave exposure in absolute and relative terms, for the age cohort born in the year 2020.

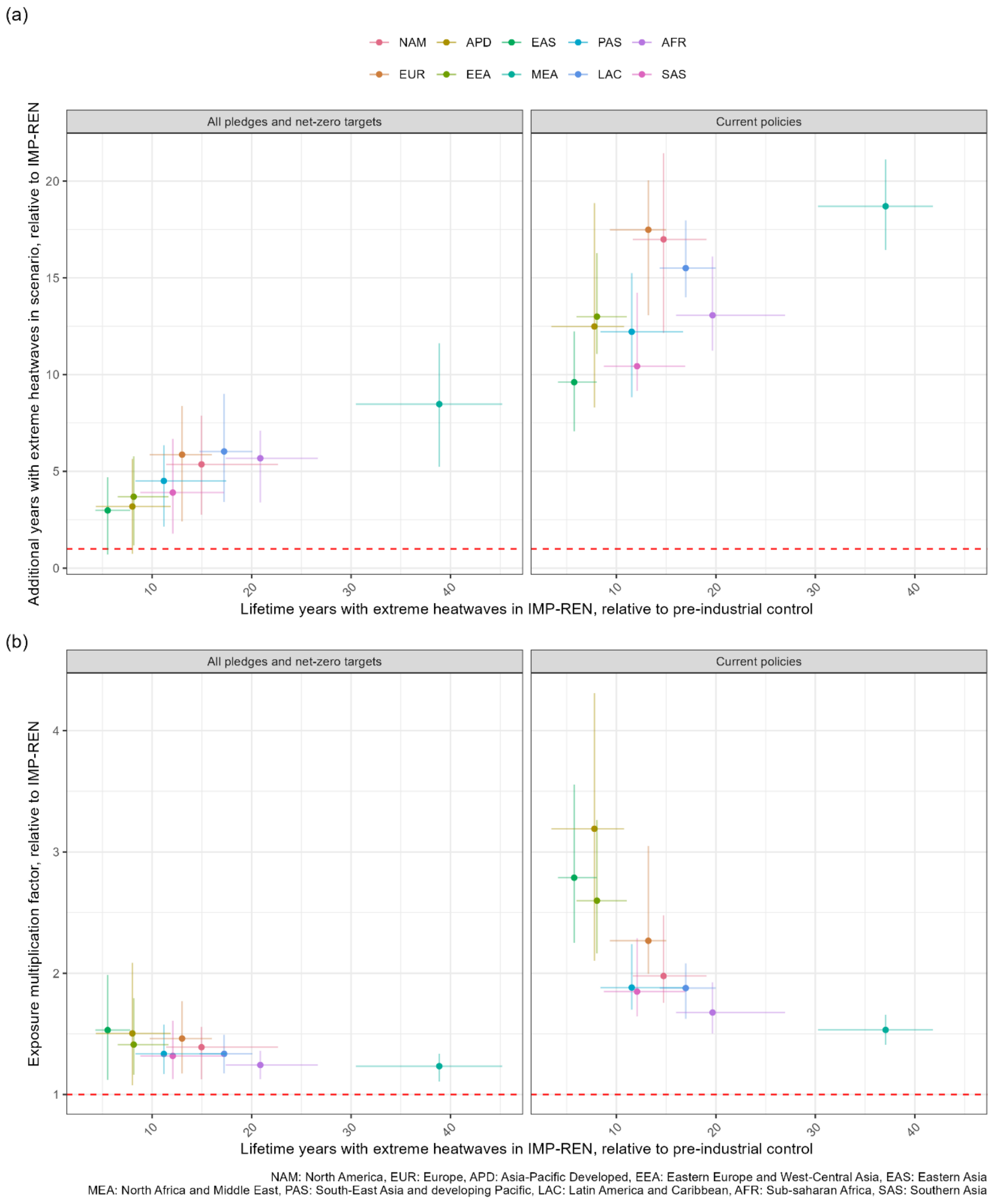

*SI Figure 5 – **(a)** Median additional years with extreme heatwaves in each scenario relative to IMP-REN (y-axis) over median additional years with extreme heatwaves in IMP-REN relative to pre-industrial control (x-axis). The error bars describe the 33rd and 66th quantiles in each case, across the full set of GCM x run x temperature response quantile combinations. **(b)** Median exposure multiplication factors (relative increase in extreme heatwaves) in each scenario relative to IMP-REN (y-axis) over median additional years with extreme heatwaves in IMP-REN relative to pre-industrial control (x-axis). The error bars describe the 33rd and 66th quantiles in each case, across the full set of GCM x run x temperature response quantile combinations.*

7. On the temperature equivalence of carbon emissions to carbon removals

When translating net-zero carbon debts into carbon drawdown obligations, we make the implicit assumption that a ton of CO_2 permanently removed will have an equivalent cooling effect to the warming effect of a ton of CO_2 emitted (for further reading on this topic, see for example (3–7)). To test the implications of this assumption here, we run CO_2 emissions and removals experiments in FaIR (8), finding an approximate 1:1 long-term temperature equivalence between gross carbon emissions prior to net-zero and net-negative permanent carbon sequestration thereafter for scenarios that limit warming to 1.5°C with no or limited overshoot, with a 25% uncertainty in either direction. While the work shown here provides an indication of robustness in translating net-zero carbon debts to carbon drawdown obligations, we note the need for further research to resolve the issues of co-emitted non-CO_2 GHGs that affect peak warming, local aerosol pollution and the uncertain hysteresis of regional climate impacts following peak cumulative carbon emissions (9–12). Furthermore, there is a possibility that uncertainties are greater for scenarios at higher warming levels (5).

We take the 97 Category 1 (C1) scenarios that limit warming to 1.5°C with low or no overshoot from the IPCC AR6 WGIII Scenario Database (13, 14). Emissions time series run to 2100. Our goal is to determine whether cooling beyond the point at which net zero CO_2 emissions are achieved and negative emissions commence, on a degree per cumulative net carbon removal measure, is equivalent to the degree warming per cumulative carbon emitted before net zero.

A necessary condition for this analysis is that the scenarios reach and pass through net zero to net negative CO_2 emissions before 2100. Three out of the 97 scenarios do not reach net zero so are excluded from the analysis. The remaining 94 scenarios are run through FaIR v2.2 using the same calibration of FaIR as noted earlier (calibration v1.4.1, 15).

We run these 94 emissions scenarios in FaIR using only the CO_2 emissions from each C1 scenario, ignoring non-CO_2 forcers. This allows isolation of the relevant carbon budget metrics without conflation by other climate-relevant species. The 94 scenarios are run from 1750 to 2100, using historical emissions up until 2014 and each C1 scenario's projected emissions from 2015. The FaIR calibration contains 841 ensemble members that span the full range of observed and assessed climate system uncertainty (for example, 90% of the distribution

having an equilibrium climate sensitivity between 2–5°C, following the *very likely* range assessed in (16)). Full details of the calibration and constraining mechanism are given in (15).

To estimate equivalence of gross carbon emissions to net-negative carbon removals, we separate the pre-net zero and post-net zero phases in each scenario and FaIR ensemble member. We define the warming phase up until net zero as the transient climate response to cumulative CO_2 emissions (TCRE) on the upward branch, $\mathrm{TCRE_{up}}$, as

$$\mathrm{TCRE_{up}} = \frac{T_{\mathrm{nz}}}{C_{\mathrm{nz}}} \qquad (3)$$

where T_{nz} is the temperature change from 1750 to the year of net zero in the scenario and C_{nz} is the cumulative CO_2 emissions from 1750 to the year of net zero. This value can be somewhat influenced by the zero emissions commitment (ZEC), defined as the residual temperature change after net zero (17), if ZEC is strongly positive or negative. As these pathways tend to approach net zero gradually, a negative ZEC can manifest itself by temperatures peaking and declining before net zero is reached (18). If ZEC is strongly positive, approaching net zero does little to slow down the rise in temperature that continuing along a TCRE path would predict.

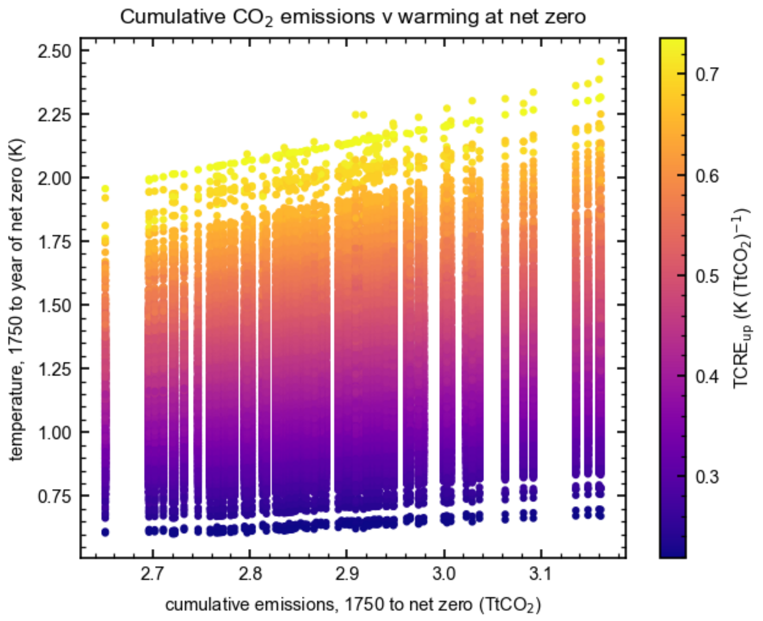

Figure S12 – Linear relationships between cumulative positive emissions at the point of net zero (C_{nz}) and temperature change at the point of net zero (C_{nz}) from 94 C1 scenarios for a set of 841 ensemble members run in FaIR with only CO2 emissions. The slope of each of the regression lines for the 841 ensemble members defines $TCRE_{up}$, which dictates the colour of the points.

Firstly, we verify that our definition of $\mathrm{TCRE_{up}}$ satisfies the usual criterion of TCRE, which is that temperature change increases linearly with cumulative CO_2 emitted. SI Figure 12 shows the plot of cumulative CO_2 emissions C_{nz} against temperature change T_{nz} for the 94 scenarios and 841 FaIR ensemble members (79054 total runs). The model runs cluster in vertical bands along the *x*-axis, as C_{nz} can take on one of 94 values corresponding to each scenario. For each scenario, the spread of values in the *y*-direction shows the spread in warming T_{nz} (841 points per band, related to climate system uncertainty) for each scenario. The points are colored according to $\mathrm{TCRE_{up}}$ in each FaIR ensemble member, demonstrating firstly the expected linearity and secondly that ensemble members with higher $\mathrm{TCRE_{up}}$ warm more for the same emissions scenario.

The temperature change between the year of net zero and 2100 as a function of cumulative net CO_2 removed is more directly influenced by ZEC than $\mathrm{TCRE_{up}}$. For example, if ZEC is large and positive, more net carbon removal is required to oppose this warming tendency than if ZEC was zero. Independently of ZEC, the effect of net-negative carbon removals on temperature change may not be equivalent to that of gross emissions. Assuming that there is also a linear change in temperature per ton CO_2 removed – a cooling effect rather than a warming effect – we denote this quantity $\mathrm{TCRE_{down}}$. To attempt to separate $\mathrm{TCRE_{down}}$ from ZEC, we propose a "Gregory" style regression of net carbon removals against post-net-zero temperature change in the 94 C1 scenarios run in FaIR (SI Figure 13), both measured from the year of net zero to 2100. This is inspired by the Gregory method of estimating climate sensitivity and effective radiative forcing from Earth System Model runs (Gregory et al., 2004). We indeed find a linear relationship between cumulative post-net-zero removals and post-net-zero temperature change (correlation coefficient r>.85 for every FaIR ensemble member and >.97 for 823 out of 841). In SI Figure 14 each vertical band again relates to one of 94 IAM scenarios and the spread across the *y*-axis relates to the climate system uncertainty in the FaIR ensemble. We assert that the slope of the regression line of each FaIR ensemble member can be interpreted as $\mathrm{TCRE_{down}}$, and the intercept of each regression line at zero cumulative emissions (*x*=0) as some measure of ZEC.

Figure S14 also hints at a correlation between $\mathrm{TCRE_{up}}$ and $\mathrm{TCRE_{down}}$, in which the lines traced by yellow and orange coloured dots (high $\mathrm{TCRE_{up}}$) have the steepest gradient (and

purple dots where low $\mathrm{TCRE_{up}}$ have the shallowest gradient representing low $\mathrm{TCRE_{down}}$), whereas there is a wide spread in the distribution of the lines with no consistent colour gradient along the *y*-axis representing a spread in ZEC estimates that are relatively uncorrelated with $\mathrm{TCRE_{up}}$.

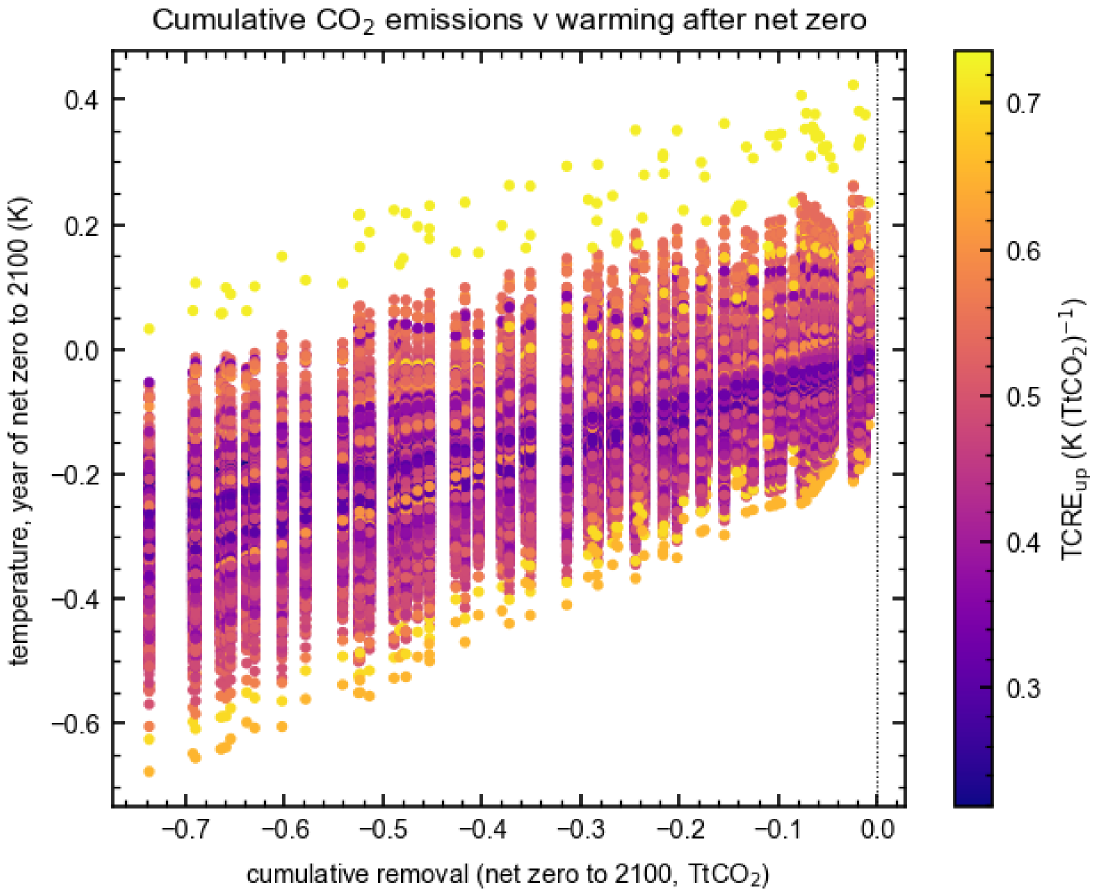

Figure S14 – Scatterplot showing the post-net-zero temperature change (y-axis) in relation to post-net-zero CO2 removals (x-axis). Points are coloured by the $TCRE_{up}$ (eq. 1) derived in each ensemble member.

Investigating this further, we indeed observe a positive, heteroskedastic correlation between $\mathrm{TCRE_{up}}$ and $\mathrm{TCRE_{down}}$ (Figure S15). We use the distribution of the ratios of $\mathrm{TCRE_{down}}$ to $\mathrm{TCRE_{up}}$ to define our measure of the efficacy of carbon removals. In this FaIR ensemble, the median value is 1.05 with a 5th-95th percentile spread of 0.76-1.25. Thus in the ensemble median, carbon removals are slightly more effective at cooling than emissions are at warming. However, taking a precautionary approach and noting the median is close to unity, we determine that the **uncertainty in efficacy of removals compared to emissions is 1, with an**

uncertainty of ±0.25. This is the range we consider as the long-term uncertainty in translating carbon debt accrual to carbon drawdown obligations due to geophysical considerations.

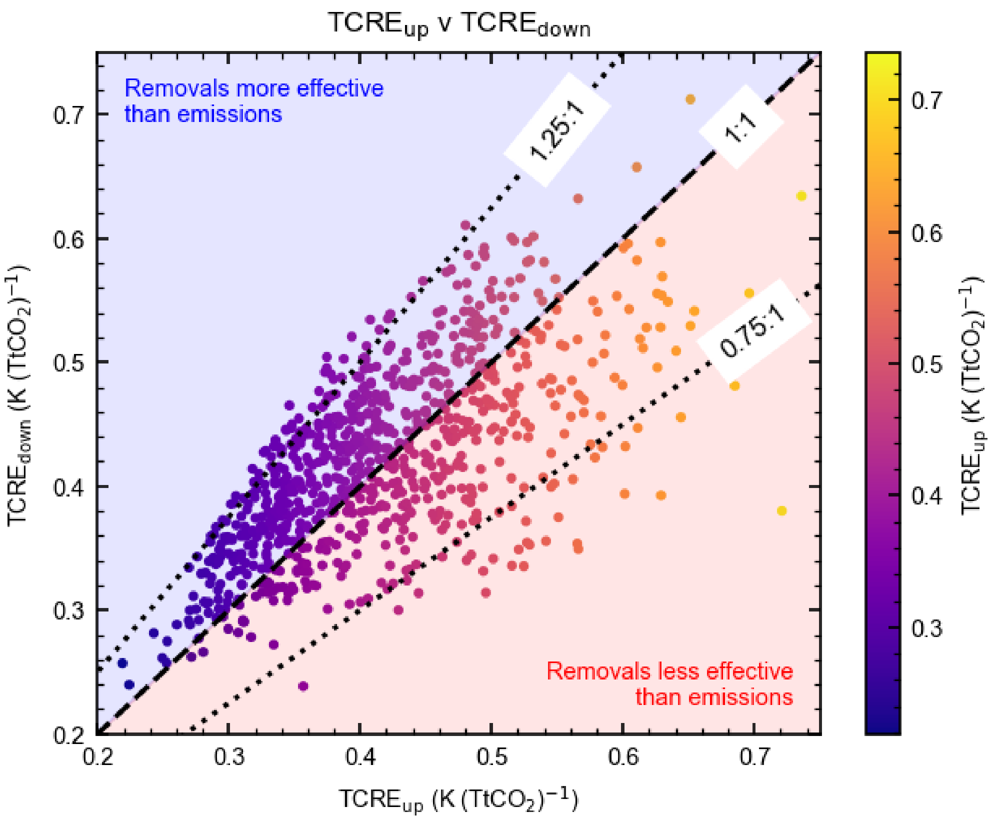

Figure S15 – Relationship between $TCRE_{up}$ and $TCRE_{down}$ in the 841 FaIR ensemble members for C1 scenarios. Below the 1:1 line (red shading), carbon removals are less effective at cooling than emissions are at warming. The blue shaded region above the 1:1 line shows the opposite. Points are coloured according to the underlying TCREup.

While our calculated "ZEC" from the regression intercepts also influences the post-net-zero cooling, the median (5th and 95th percentiles) of our ZEC distribution is -0.02 (-0.10 to +0.10)°C. Thus, to first order, we can claim that ZEC is approximately zero on the timescales and levels of emissions relevant to our study. This means that we can exclude ZEC from the calculation of equivalence and use the $\mathrm{TCRE_{down}}/\mathrm{TCRE_{up}}$ ratio as an estimate of removal equivalence.

We now further justify that our methods of derivation are appropriate. To show that our regression-derived "ZEC" is an appropriate measure of the calculation of ZEC using more standardized experiments, we compare our regression-derived ZEC to the ZEC_{50} (temperature change 50 years after zero emissions) from flat10-MIP (7) for the same ensemble of FaIR (calibration v1.4.1) in Figure S16. The time horizon of our ZEC measure depends on the number of years between each scenario reaching net zero and 2100. For C1 scenarios, the

median net zero date is between 2050 and 2055 and 90% of scenarios have net zero dates between 2035 and 2070 (IPCC Working Group III SPM). Therefore, there is a 47.5 ± 17.5 year difference between net zero and 2100 in C1 scenarios, and our ZEC measure can be approximately compared to ZEC_{50} from idealized simulations.

The flat10-MIP measure of ZEC_{50} is derived from running FaIR for 100 years with 10 GtC emissions per year, suddenly dropping emissions to zero, running a further 50 years, and reporting the temperature difference in year 150 relative to year 100. SI Figure 16 shows that there is a strong positive correlation between our regression-derived ZEC and ZEC_{50} from flat10-MIP. This first demonstrates that our regression-derived quantity is an appropriate definition of a zero emissions commitment. However, our regression-derived ZEC tends to be smaller in absolute magnitude than an idealized ZEC_{50}. This is due to the differences in the way that the estimates are constructed. For our purposes, the regression is more appropriate since the C1 scenarios describe internally consistent pathways from IAMs, and are therefore more representative of the real world or any realistic ambitious scenario that would get close to limiting warming to 1.5°C. As previously discussed, a smaller spread in ZEC is useful as it influences the $\mathrm{TCRE}_{\mathrm{down}}/\mathrm{TCRE}_{\mathrm{up}}$ ratio less, which is another factor to prefer the regression estimates.

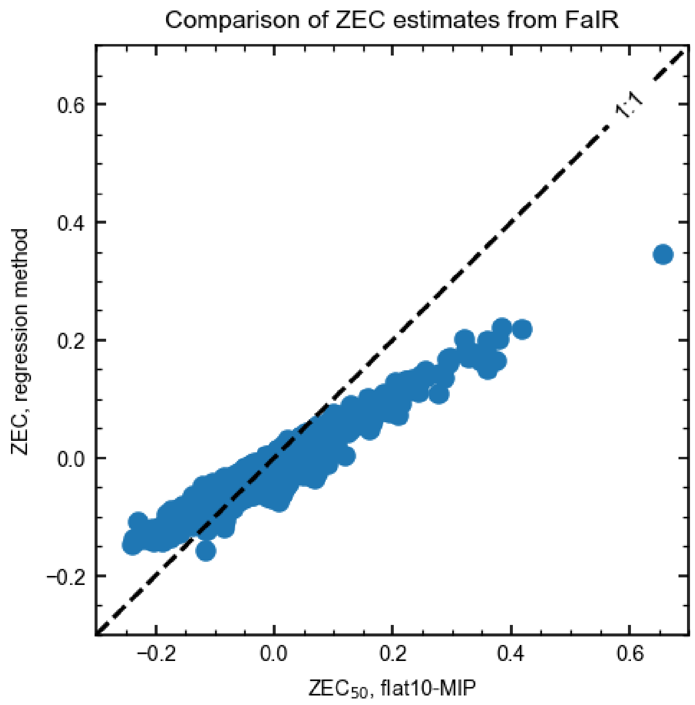

Figure S16 – Comparison of ZEC determined from the regression method in this study and from flat10-MIP for ZEC50 (Sanderson et al., submitted) for the same FaIR ensemble.

For further justification we report key statistics from the ensembles of flat10-MIP and the regression method in Table S1. The ensemble medians for ZEC (C1 regression) and ZEC_{50} (flat10-MIP), $\mathrm{TCRE_{up}}$ (C1 regression) and TCRE (flat10-MIP), $\mathrm{TCRE_{down}}$, and $\mathrm{TCRE_{down}}/\mathrm{TCRE_{up}}$ are similar for the two measures.

In flat10-MIP, TCRE is determined as the temperature change after 100 years of constant 10 GtC emissions (cumulative emissions 1 TtC) and is comparable to $\mathrm{TCRE_{up}}$. $\mathrm{TCRE_{down}}$ is calculated as

$$\mathrm{TCRE_{down,flat10}} = \frac{T_{300} - T_{200}}{C_{300} - C_{200}} = \frac{T_{300} - T_{200}}{-1\ [\mathrm{TtC}]} \quad (4)$$

from the experiment flat10-CDR (7) . In this experiment, emissions go up at 10 GtC yr^{-1} from year 0 to year 100, decline in a straight line to -10 Gt yr^{-1} in year 200 (passing net zero in year 150), and maintaining constant -10 GtC yr^{-1} until year 300. The subscripts 200 and 300 in eq. (2) refer to the temperatures T or cumulative emissions C at these years. At year 300, cumulative emissions are back to zero and at year 200 the temperature is at the value of TCRE (at year 100) plus any adjustment for ZEC100, which again is close to zero in the ensemble median for this calibration of FaIR.

Table S1 – Comparison of carbon cycle metrics between idealized experiments in flat10-MIP and the C1 scenario regression. In the first column Quantities in parenthesis show the flat10 metric being compared

	**flat10-MIP**			**C1 scenario regression**		
	5th perc.	Median	95th perc.	5th perc.	Median	95th perc.
**ZEC (ZEC_{50}), K**	-0.15	-0.02	+0.20	-0.10	-0.02	+0.10
**$TCRE_{up}$ (TCRE), K TtC^{-1}**	1.07	1.46	1.94	1.08	1.49	2.09
**$TCRE_{down}$, K TtC^{-1}**	1.08	1.52	2.16	1.14	1.54	2.02
**$TCRE_{down}/TCRE_{up}$ ($TCRE_{down}$/TCRE)**	0.96	1.04	1.15	0.76	1.05	1.25

Since $\mathrm{TCRE_{down,flat10}}$ is calculated at 1000 GtC cumulative emissions after 100 years of net negative removals at 10 GtC yr^{-1}, five times larger than the largest net negative removal in C1 scenarios and over a longer time horizon, we again claim the regression method based on C1 scenarios is most appropriate for our conclusions. $\mathrm{TCRE_{down}}/\mathrm{TCRE_{up}}$ is possibly over-constrained in flat10-MIP, again as the experiments permit a longer time horizon for the

metrics in the numerator and denominator to emerge. As the spread in ZEC is wider in flat10-MIP than our regression method but the spread in ratio in $\mathrm{TCRE_{down}}/\mathrm{TCRE_{up}}$ is smaller, we can interpret the regression method as repartitioning some of the warming and cooling in the pre- and post-net-zero phases into TCRE rather than ZEC. The qualitative agreement with flat10-MIP-derived metrics is validation that the regression method is useful.

References

1. M. W. Jones, *et al.*, National contributions to climate change due to historical emissions of carbon dioxide, methane, and nitrous oxide since 1850. *Sci Data* **10**, 155 (2023).

2. M. Li, S. Pelz, R. Lamboll, C. Wang, J. Rogelj, A principle-based framework to determine countries' fair warming contributions to the Paris Agreement. *Nat Commun* **16**, 1043 (2025).

3. A. H. MacDougall, K. Zickfeld, R. Knutti, H. D. Matthews, Sensitivity of carbon budgets to permafrost carbon feedbacks and non-CO_2 forcings. *Environ. Res. Lett.* **10**, 125003 (2015).

4. K. Zickfeld, A. H. MacDougall, H. D. Matthews, On the proportionality between global temperature change and cumulative CO_2 emissions during periods of net negative CO_2 emissions. *Environ. Res. Lett.* **11**, 055006 (2016).

5. K. B. Tokarska, K. Zickfeld, J. Rogelj, Path Independence of Carbon Budgets When Meeting a Stringent Global Mean Temperature Target After an Overshoot. *Earth's Future* **7**, 1283–1295 (2019).

6. K. Zickfeld, D. Azevedo, S. Mathesius, H. D. Matthews, Asymmetry in the climate–carbon cycle response to positive and negative CO2 emissions. *Nat. Clim. Chang.* **11**, 613–617 (2021).

7. B. M. Sanderson, *et al.*, flat10MIP: An emissions-driven experiment to diagnose the climate response to positive, zero, and negative CO2 emissions. [Preprint] (2024). Available at: https://egusphere.copernicus.org/preprints/2024/egusphere-2024-3356/ [Accessed 17 November 2024].

8. C. Smith, *et al.*, fair-calibrate v1.4.1: calibration, constraining, and validation of the FaIR simple climate model for reliable future climate projections. *Geosci. Model Dev.* **17**, 8569–8592 (2024).

9. A. H. MacDougall, J. Mallett, D. Hohn, N. Mengis, Substantial regional climate change expected following cessation of CO_2 emissions. *Environ. Res. Lett.* **17**, 114046 (2022).

10. K. Zickfeld, *et al.*, Net-zero approaches must consider Earth system impacts to achieve climate goals. *Nat. Clim. Chang.* **13**, 1298–1305 (2023).

11. P. Pfleiderer, C.-F. Schleussner, J. Sillmann, Limited reversal of regional climate signals in overshoot scenarios. *Environ. Res.: Climate* **3**, 015005 (2024).

12. C.-F. Schleussner, *et al.*, Overconfidence in climate overshoot. *Nature* **634**, 366–373 (2024).

13. E. Byers, *et al.*, AR6 Scenarios Database. Zenodo. https://doi.org/10.5281/ZENODO.5886911. Deposited 9 November 2022.

14. IPCC, Ed., “Mitigation Pathways Compatible with Long-term Goals” in *Climate Change 2022 - Mitigation of Climate Change*, 1st Ed., (Cambridge University Press, 2023), pp. 295–408.

15. C. Smith, *et al.*, fair-calibrate v1.4.1: calibration, constraining and validation of the FaIR simple climate model for reliable future climate projections. [Preprint] (2024). Available at: https://egusphere.copernicus.org/preprints/2024/egusphere-2024-708/ [Accessed 21 October 2024].

16. P. Forster, *et al.*, “The Earth’s Energy Budget, Climate Feedbacks, and Climate Sensitivity” in *Climate Change 2021: The Physical Science Basis*, V. Masson-Delmotte, *et al.*, Eds. (Cambridge University Press, 2021), pp. 923–1054.

17. A. H. MacDougall, *et al.*, Is there warming in the pipeline? A multi-model analysis of the Zero Emissions Commitment from CO₂ *Biogeosciences* **17**, 2987–3016 (2020).

18. C. D. Koven, B. M. Sanderson, A. L. S. Swann, Much of zero emissions commitment occurs before reaching net zero emissions. *Environ. Res. Lett.* **18**, 014017 (2023).
